# Preliminary Study of a 1,5-Benzodiazepine-Derivative Labelled with Indium-111 for CCK-2 Receptor Targeting

**DOI:** 10.3390/molecules26040918

**Published:** 2021-02-09

**Authors:** Marco Verona, Sara Rubagotti, Stefania Croci, Sophia Sarpaki, Francesca Borgna, Marianna Tosato, Elisa Vettorato, Giovanni Marzaro, Francesca Mastrotto, Mattia Asti

**Affiliations:** 1 Department of Pharmaceutical Sciences, University of Padova, via Marzolo 5, 35131 Padova, Italy; marco.verona@phd.unipd.it (M.V.); francesca.borgna@psi.ch (F.B.); elisa.vettorato@unipd.it (E.V.); giovanni.marzaro@unipd.it (G.M.); francesca.mastrotto@unipd.it (F.M.); 2 Radiopharmaceutical Chemistry Section, Nuclear Medicine Unit, AUSL-IRCCS di Reggio Emilia, Viale Risorgimento 80, 42122 Reggio Emilia, Italy; sara.rubagotti@libero.it; 3 Clinical Immunology, Allergy, and Advanced Biotechnologies Unit, Diagnostic Imaging and Laboratory Medicine Department, AUSL-IRCCS di Reggio Emilia, Viale Risorgimento 80, 42122 Reggio Emilia, Italy; Stefania.Croci@ausl.re.it; 4 Bioemtech, Lefkippos Attica Technology Park N.C.S.R. “DEMOKRITOS” Patr. Gregoriou E & 27 Neapoleos Str. Ag. Paraskevi, 15341 Athens, Greece; ssarpaki@bioemtech.com; 5 Department of Chemical Sciences, University of Padova, via Marzolo 1, 35131 Padova, Italy; marianna.tosato@phd.unipd.it; 6 Legnaro National Laboratories, Italian Institute of Nuclear Physics, Viale dell’Università 2, 35020 Legnaro (Padova), Italy

**Keywords:** cholecystokinin-2 receptor, indium-111 labelling, nastorazepide, radiopharmaceuticals

## Abstract

The cholecystokinin-2 receptor (CCK-2R) is overexpressed in several human cancers but displays limited expression in normal tissues. For this reason, it is a suitable target for developing specific radiotracers. In this study, a nastorazepide-based ligand functionalized with a 1,4,7,10-tetraazacyclododecane-1,4,7,10-tetraacetic acid (DOTA) chelator (IP-001) was synthesized and labelled with indium-111. The radiolabeling process yielded >95% with a molar activity of 10 MBq/nmol and a radiochemical purity of >98%. Stability studies have shown a remarkable resistance to degradation (>93%) within 120 h of incubation in human blood. The in vitro uptake of [^111^In]In-IP-001 was assessed for up to 24 h on a high CCK-2R-expressing tumor cell line (A549) showing maximal accumulation after 4 h of incubation. Biodistribution and single photon emission tomography (SPECT)/CT imaging were evaluated on BALB/c nude mice bearing A549 xenograft tumors. Implanted tumors could be clearly visualized after only 4 h post injection (2.36 ± 0.26% ID/cc), although a high amount of radiotracer was also found in the liver, kidneys, and spleen (8.25 ± 2.21%, 6.99 ± 0.97%, and 3.88 ± 0.36% ID/cc, respectively). Clearance was slow by both hepatobiliary and renal excretion. Tumor retention persisted for up to 24 h, with the tumor to organs ratio increasing over-time and ending with a tumor uptake (1.52 ± 0.71% ID/cc) comparable to liver and kidneys.

## 1. Introduction

Receptor-specific targeting ligands were recently harnessed to transport and selectively deliver cytotoxic payloads to cancer tissues in vivo. Payloads may vary from chemotherapeutics to radionuclides and are usually strongly tethered to a chemically modified endogenous ligand or a synthetic agent that expresses a high affinity for the selected receptors [1,2]. Focusing on radioactive cargos, the use of metal radionuclides allows for the achievement of both diagnostic and therapeutic purposes since the same targeting vector can be labelled with metals exhibiting different emissions but similar or equal chemical features. A stable bond between a radiometal and ligand is usually provided by using proper bifunctional chelators, i.e., chemical compounds covalently bind to the backbone of the targeting vector and are able to form thermodynamically and kinetical stable complexes with the metal [3,4].

Among target receptors, cholecystokinin-2 receptor (CCK-2R) is a G-protein-coupled receptor that is normally expressed in the central nervous system and gastric mucosa, where it conveys a regulatory function. Endogenous ligands for CCK-2R are low molecular-weight peptides mainly synthesized in the central nervous system and the gastrointestinal tract such as gastrin and cholecystokinin. CCK-2R is also overexpressed in various human cancers (e.g., lung, medullary thyroid, pancreatic, colon, and gastrointestinal stromal tumors), where it stimulates cell growth, migration, and tumor metastasis, but it displays limited expression in other normal tissues [5,6,7,8]. For this reason, CCK-2R is a suitable target for functional imaging and therapy with radiopharmaceuticals, and its potential has been largely explored in the last few years by using various radiolabeled derivatives of its endogenous activators [9]. However, the use of peptide-based ligands still raises some concerns because derivatives exhibiting the highest tumor uptake are also characterized by high retention in the kidneys. Conversely, radiolabeled peptides that display low accumulation in kidneys show little retention in tumor tissues as well. Secondly, being the peptide chains prone to degradation by endogenous peptidases and physiological oxidation on methionine residue, the affinity for receptors can be thwarted during circulation, thus resulting in a suboptimal distribution with a consequent accumulation of high radioactive doses in healthy tissues. Finally, the use of agonist such as CCK- or gastrin-derivatives may activate the receptor signal stimulating the growth, proliferation, and survival of cancer cells [10,11]. As a consequence, the development of a peptide-based CCK-2R-targeting molecule for radiotherapeutic applications has been hampered so far, although many improvements have been obtained over the years through several interesting approaches [12,13,14].

An alternative path to outperform these issues could be achieved by labelling antagonist ligands based on small organic molecules rather than amino acid sequences. As opposed to agonists, antagonists do not activate the signaling cascade when bound to the receptor, and a non-peptide-based structure should provide a higher resistance to enzymatic degradation. Nastorazepide (Z-360) is a selective 1,5-benzodiazepine-derivative CCK-2 receptor antagonist with potential antineoplastic activity. Z-360 binds to CCK-2R, leading to the avoidance of its activation with sub-nanomolar affinity and high selectivity, in contrast to CCK-1R (K_d_ = 0.47 nmol/L; selectivity relative to CCK-1R = 672) [15].

The usefulness of Z-360 as directing moiety for the preparation of radiopharmaceutical has already been demonstrated in the literature. For example, a series of nastorazepide-based derivatives have been synthesized and tethered to a N_3_S- or N_4_-system bifunctional chelator through different spacers [16,17]. The introduction of these chelators provided a suitable moiety for technetium-99m (t_1/2_ = 6 h; E_γ_ = 140 keV) complexation, while appropriate spacers were introduced to optimize affinity and water solubility for CCK-2R. As a result, it has been demonstrated that Z360-based radiopharmaceuticals are valuable tools that are able to yield high-resolution images of CCK-2R-expressing tumors, especially at longer acquisition times.

Based on these groundbreaking results, in the present study, a 1,5-benzodiazepine-based ligand functionalized with a 1,4,7,10-tetraazacyclododecane-1,4,7,10-tetraacetic acid (DOTA) chelator was synthesized in order to yield a precursor with theragnostic potential. DOTA is an almost universal chelator that is able to complex (with high stability) several metal radionuclides currently applied in clinical practice such as gallium-68 (t_1/2_ = 68 m; E_β+_ = 0.89 MeV), indium-111 (t_1/2_ = 2.8 d; E_γ_ = 173 and 247 keV), lutetium-177 (t_1/2_ = 6.7 d; E_β(max)_ = 497, 384, and 176 keV; E_γ_  =  113 and 208 keV), just to mention a few [18]. Herein, the labelling of the DOTA-functionalized nastorazepide derivative was carried out with indium-111. Indium-111 was preferred among the other diagnostic radionuclides for its established use in pre-clinical and clinical studies, as well as its half-life that is suitable for long-term evaluations. In fact, in our explorative experiment, indium-111 was particularly adapted to shed light on the uptake and pharmacokinetic of the radiotracer for several hours, giving better insights into the structure–metabolism relationship.

## 2. Results

### 2.1. Synthesis of the Ligand (IP-001)

**Scheme 1:**
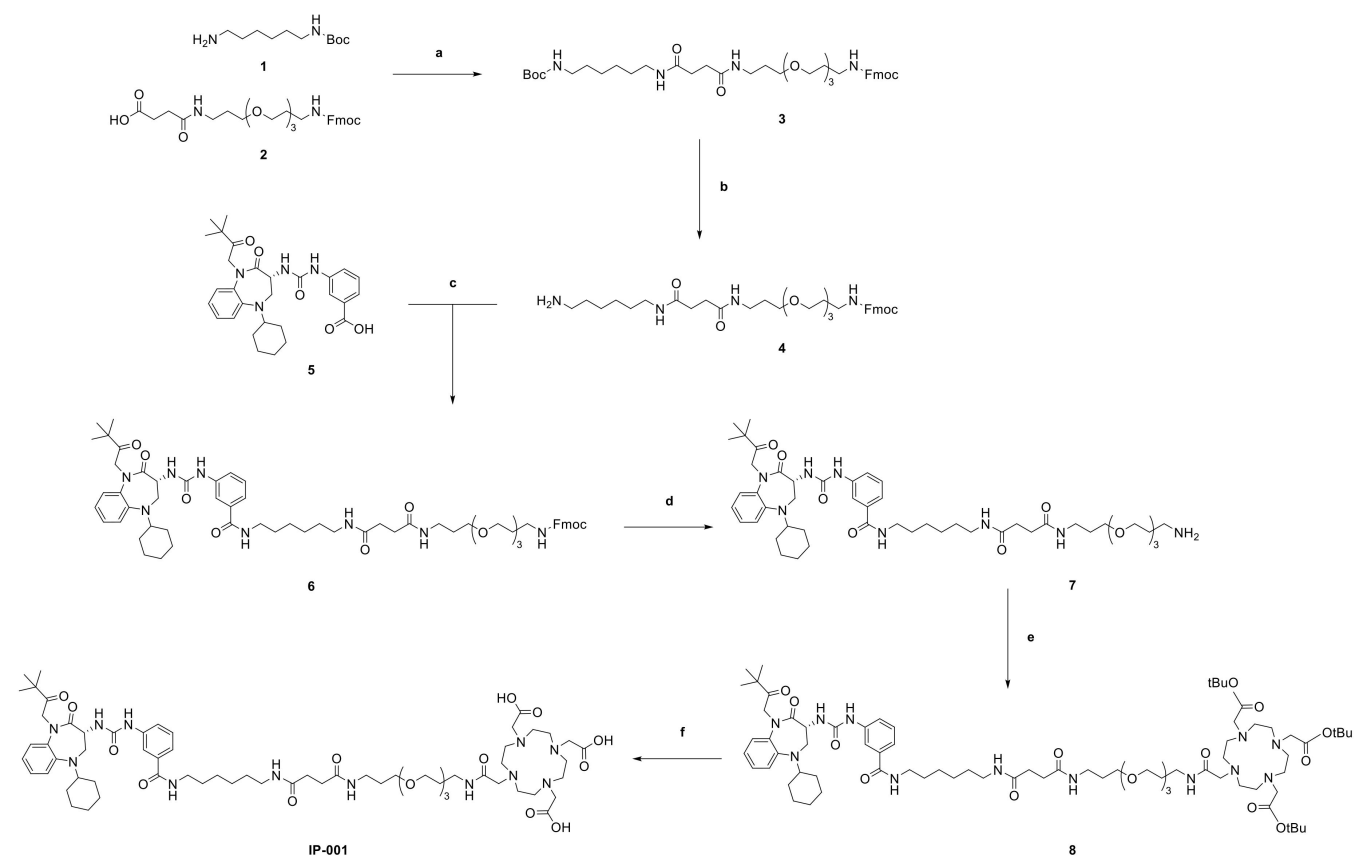
Reaction conditions: a: **2**, 1,1′-carbonyldiimidazole (CDI), MeCN; triethylamine (TEA), **1**, MeCN. 1 h (reflux); 2 h (RT). b: trifluoroacetic acid (TFA) (30%), dichloromethane (DCM) (0 °C). 1 h (RT). c: **5**, CDI, MeCN; TEA, **4**, MeCN. 1 h (reflux); 1 h (RT) d: Morpholine 50%, **6**, dimethylformamide (DMF) (0 °C). 1.5 h (RT). e: benzotriazol-1-yloxytris(dimethylamino)phosphonium hexafluorophosphate (BOP), TEA, 1,4,7,10-tetraazacyclododecane-1,4,7,10-tetraacetic acid (DOTA)(tBu)_3_ ester, DMF, **7**. 24 h (RT) f: **8**, TFA (30%), DCM, (0 °C). 16 h (RT).

The synthesis of the nastorazepide core (**5**) was performed as previously reported [19] with only few modifications, with an overall yield of 23%. The assemblage of the linker started with a condensation reaction between N-Boc-hexanediamine (**1**) and 1,1′-carbonyldiimidazole (CDI)-activated N-Fmoc-N″-succinyl-4,7,10-trioxa-1,13-tridecanediamine (**2**) to yield compound **3**. The selective elimination of the Boc-protecting group was then achieved through a reaction with 30% trifluoroacetic acid (TFA) in dichloromethane (DCM) to obtain the corresponding amino-derivate **4** in a quantitative yield. After the activation of the carboxylic group with CDI, nastorazepide (**5**) was condensed with the linker **4** to yield compound **6**. The fluorenylmethoxycarbonyl (Fmoc)-protecting group was eliminated by a reaction with 50% morpholine in dimethylformamide (DMF) to obtain compound **7**, which was subsequently reacted with the DOTA(tBu)_3_ ester to give the functionalized nastorazepide-based ligand **8**. The carboxylic functions of the DOTA chelators were finally deprotected with 30% TFA in DCM to yield IP-001 (Scheme 1). The formation of the intermediate compounds was monitored by ^1^H-NMR (Figures S1–S5 in Supplementary Materials), and the final product of the reaction was also characterized by HRMS (Figures S6 and S7). The overall yield of the process was around 5%. Predictions of lipophilicity and other pharmacokinetic properties for IP-001 and Z-360 are reported in Table 1.

### 2.2. Labelling of IP-001 with Indium-111

IP-001 was labelled with a solution of indium-111 chloride in an acetate-buffered environment (pH 4.6). Incorporation was generally higher than 95% when 50 μg of precursor were used, and the total volume of the reaction was maintained at around 1 mL, achieving a molar activity of about 10 MBq/nmol. Impurities were mainly due to unlabeled indium-111 only. Further purification with solid phase extraction (SPE) enabled a radiochemical purity (RCP) higher than 99%, but the procedure was generally avoided for the in vitro and in vivo studies due to the high concentration of EtOH used in the process that may have been detrimental for cells and animals. Quality controls were performed by means of a reverse phase HPLC (R_t_: free-[^111^In]In^3+^ = 1.1 min; [^111^In]In-IP-001 = 6.0 min) and radio-TLC (thin-layer chromatography) (R_f_: free-[^111^In]In^3+^ = 0.0; [^111^In]In-IP-001 = 0.6). Paradigmatic examples of chromatograms are reported in Figures S8 and S9.

### 2.3. Stability, Serum Proteins Binding, and Lipophilicity Studies

The long-term stability of the radiotracer (up to 120 h) was tested by TLC and RP-HPLC in various media, evaluating the resistance to degradation of the non-peptide-based backbone at different pHs (NaCl 0.9% solution pH 7 and 0.1 M 4-(2-hydroxyethyl)-1-piperazineethanesulfonic acid (HEPES) pH 4) and to enzymatic cleavage (for instance, to proteases in human serum and blood). The kinetic inertness of [^111^In]In–DOTA complexes were also evaluated in presence of a 0.1 M ethylenediaminetetraacetic acid (EDTA) solution or serum proteins as competitors. All these studies revealed a high stability of the radiotracer showing a percentage of intact compound generally higher than 95% after 120 h when incubated with NaCl 0.9%, HEPES and EDTA, and after 48 h when challenged with human serum or blood (HB). Results obtained are summarized in Table 2. Amount of radiotracer bound to serum proteins was computed in the samples incubated with HB after 8, 24 and 48 h. After treatment with a MeCN/H_2_O/TFA 50/45/5 *v*/*v*/*v* solution and centrifugation, the radioactivity in the precipitate was found noteworthy being from 12 to 20% of the total. Lipophilicity calculation gave a partition coefficient (LogP) of 0.45.

### 2.4. Selection of CCK-2R-Expressing Human Cancer Cell Lines

**Figure 1:**
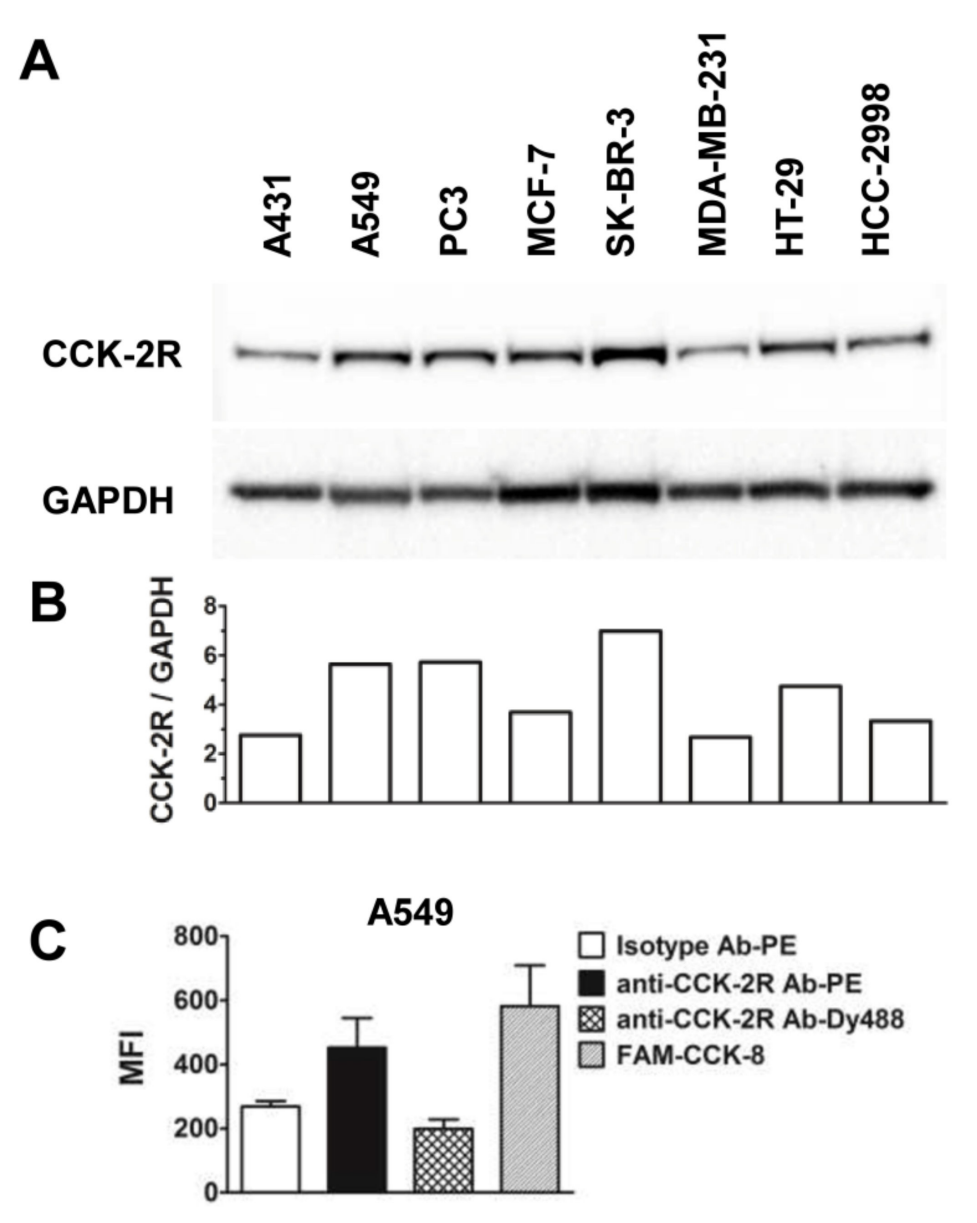
(**A**) Western blot and (**B**) quantification of the total cholecystokinin-2 receptor (CCK-2R) expression on different cell lines. GADPH served as the loading control for total proteins. (**C**) Levels of CCK-2R expression on cell surface by flow cytometry. Dead cells were removed from the analysis using LIVE/DEAD staining. The fluorescence intensity of control cells stained only with the LIVE/DEAD was subtracted. Mean fluorescence intensity plus SEM of three independent experiments is shown.

To identify the most suitable cell line for the in vitro evaluation of the radiolabeled ligand, CCK-2R protein expression was investigated in human cancer cell lines derived from tumors of different origins (non-small cell lung cancer, skin cancer, prostate cancer, breast cancer, and colon cancer). Western blot analysis revealed that CCK-2R was expressed to a certain extent by all the tested human cancer cell lines, with A549, PC3, and SK-BR-3 exhibiting the highest expression levels (Figure 1, panel A, B). Eventually, the A549 cell line was selected for the following experiments since, upon equal CCK-2R expression, it has the major advantage of ease of cultivation and efficiently forms tumors in nude mice xenograft models. Importantly, to determine whether CCK-2R was also expressed on the A549 cell surface, flow cytometry studies were performed on live cells. Cells were incubated with two different fluorescently-labelled anti-CCK-2R antibodies and, alternatively, with the fluorescent endogenous octapeptide CCK-8 (FAM-CCK-8), which binds CCK-2R with a subnanomolar affinity. Expression was confirmed, to some extent, using both antibodies and the natural ligand (Figure 1, panel C).

### 2.5. [111. In]In-IP-001 Cellular Uptake

**Figure 2:**
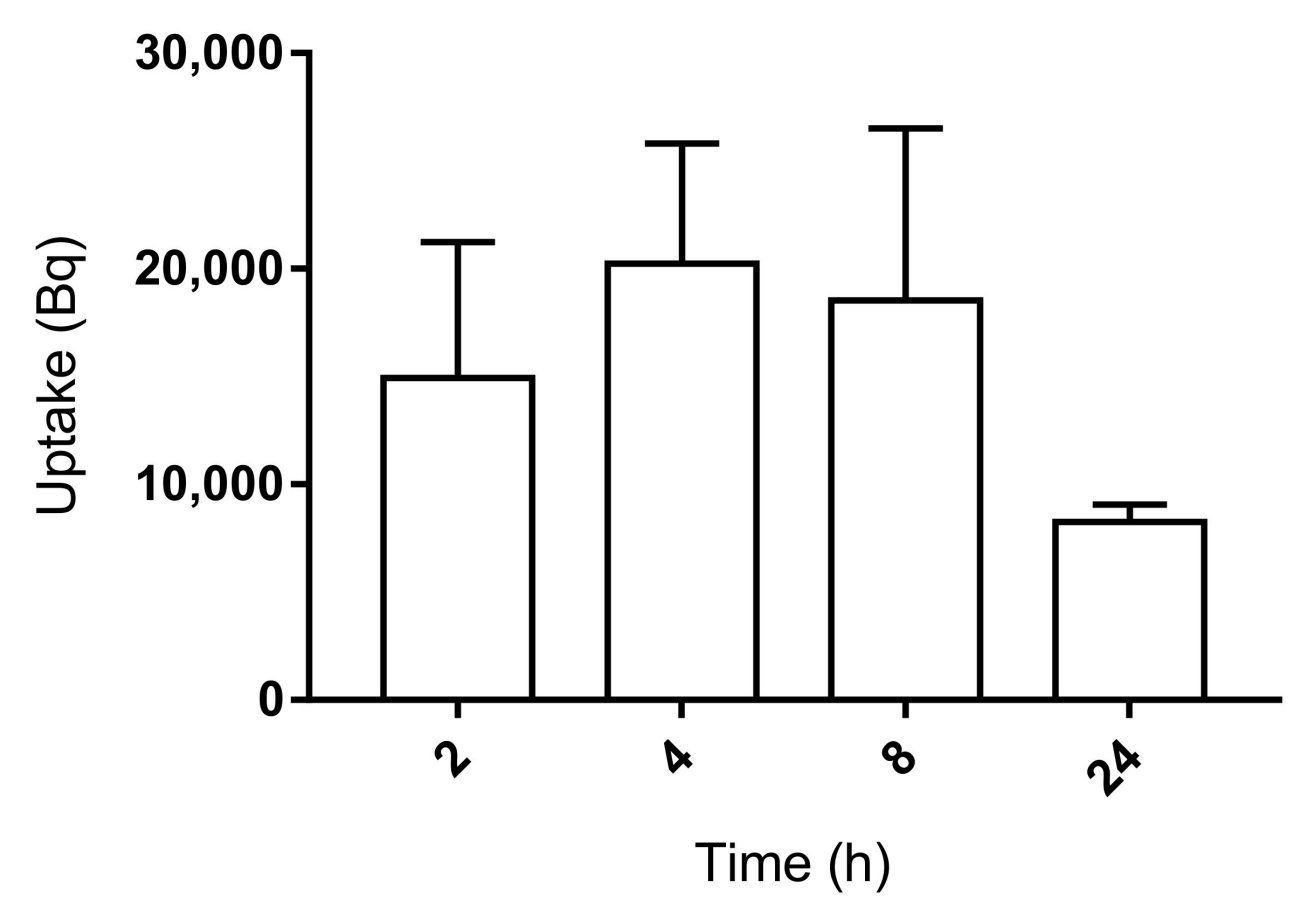
Uptake of [^111^In]In-IP-001 by A549 cells at different incubation times (*n* = 3; mean ± SD).

To study [^111^In]In-IP-001 uptake, A549 cells were incubated with 2 MBq of the radiotracer for different times of up to 24 h. Experiments were performed to gain some insights about the kinetics of the accumulation of the radiolabeled compound in vitro, with the aim to design a further and more exhaustive study in vivo. The maximal accumulation was achieved after 4 h of incubation and was around 5% (expressed as percent of the added radioactivity per million cells). After 8 h, the uptake was only slightly decreased, but it was more than halved after 24 h of incubation. Data elucidated slow kinetics regarding both the uptake and efflux of the radiotracer in vitro. The obtained results are summarized in Figure 2.

### 2.6. In Vivo Experiments

**Figure 3:**
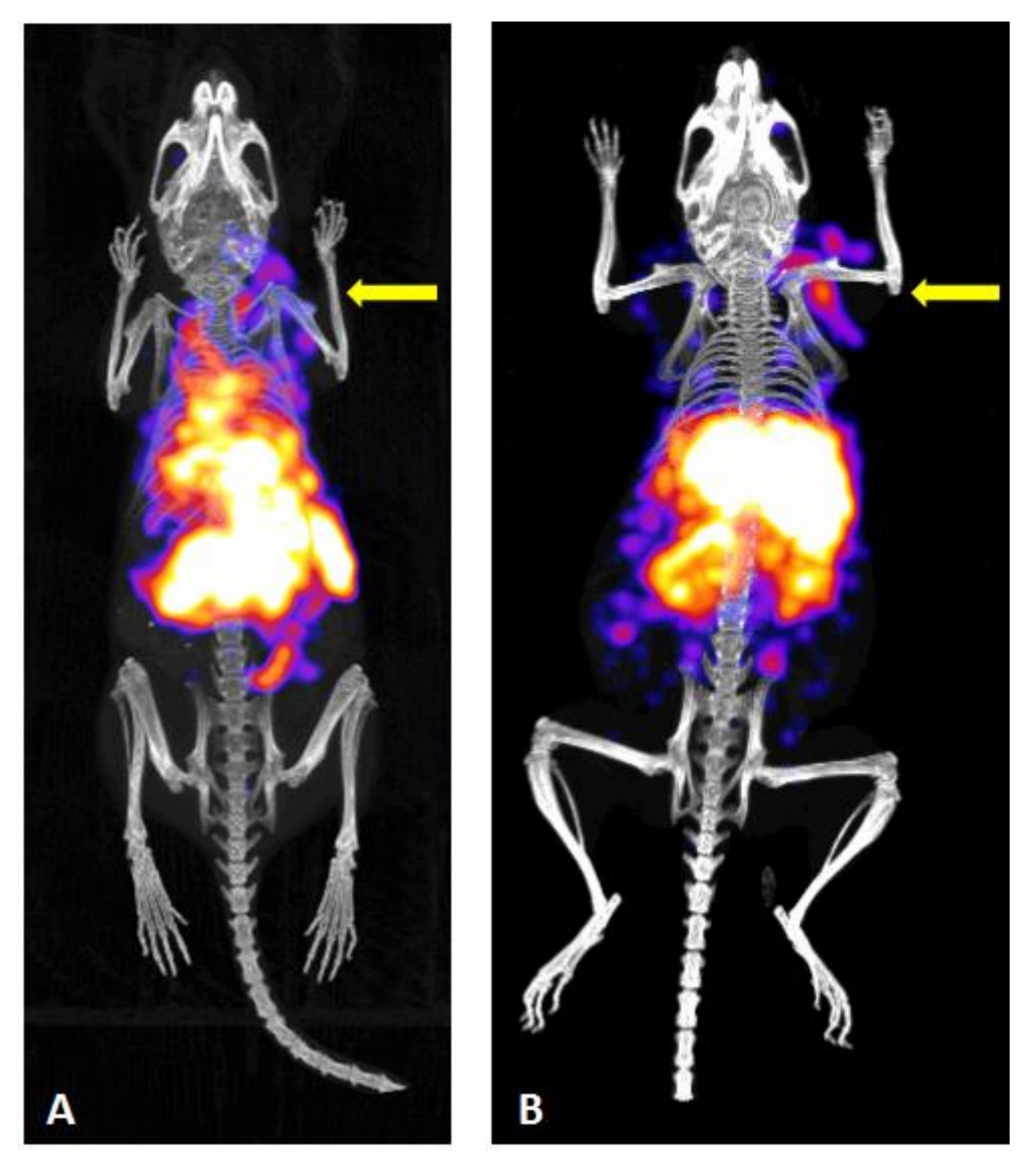
Representative single photon emission tomography (SPECT)/CT scans as maximum intensity projection (MIP) views of an A549 tumor-bearing mouse at 4 (**A**) and 24 h (**B**) post injection of about 7.4 MBq of [^111^In]In-IP-001. Tumor position in the shoulder is indicated by the arrow.

**Figure 4:**
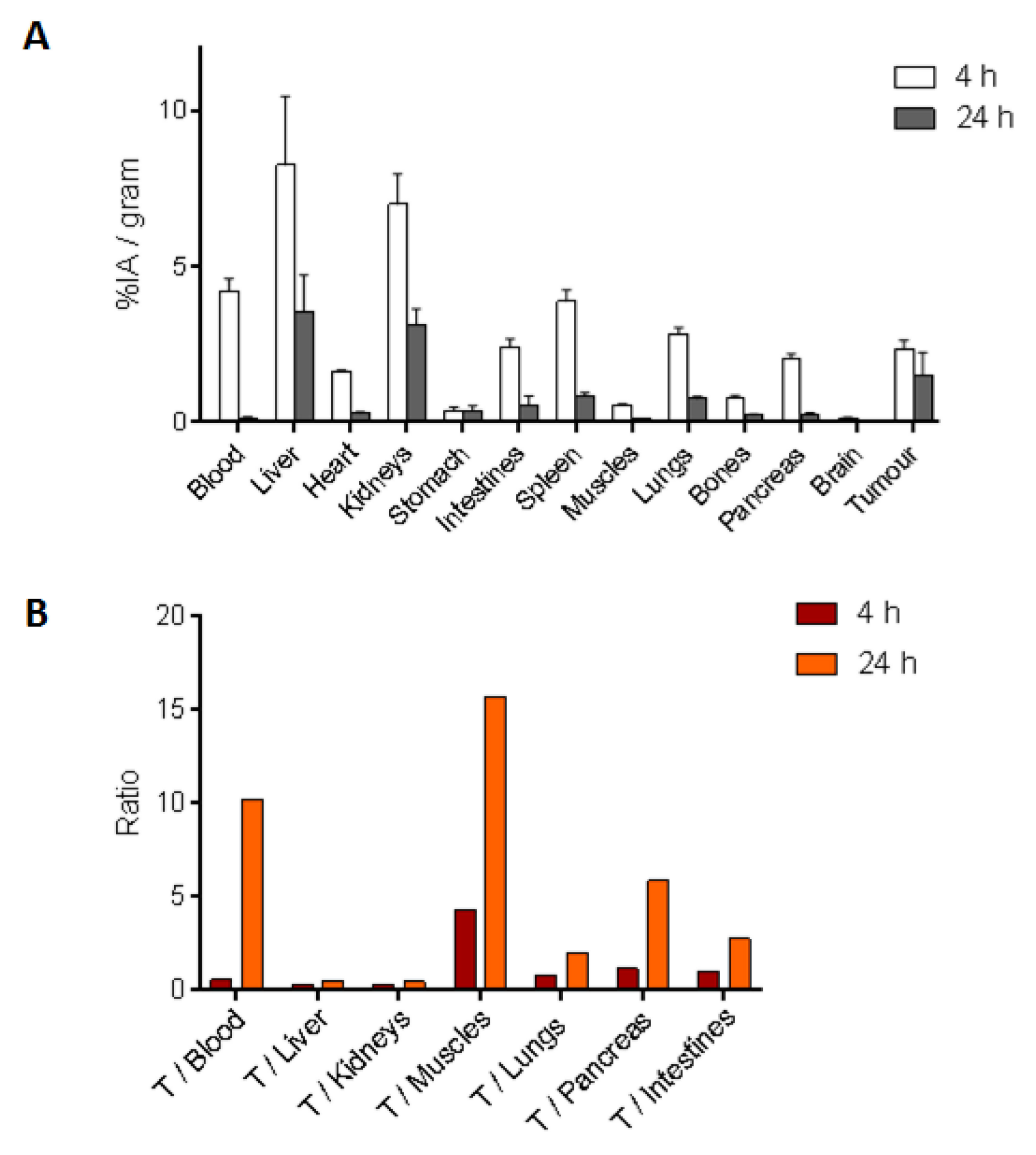
Biodistribution in multiple organs of [^111^In]In-IP-001 in A549 cancer-bearing nude mice (*n* = 3; mean ± SD) at 4 and 24 h post injection (**A**); ratios of the tumor uptake to major organs (**B**).

The A549 tumor cell line was inoculated, by subcutaneous injection, in the right shoulder of homozygous female BALB/c nude mice. The tumor was allowed to grow under observation for five weeks, and imaging procedures were started when 80% of the animals developed tumors of sufficient size (i.e., >0.5 cm). A first group of mice (*n* = 5) was intravenously injected with [^111^]In-IP-001 (2.06 nmol, 7.4 MBq), and tomographic single photon emission tomography (SPECT)/CT images were acquired at 2, 4, 8, and 24 h post injection to monitor the pharmacokinetics of the radiotracer. For blocking experiments, a second group of mice (*n* = 5) was injected with approximately the same amount of [^111^]In-IP-001 of the first group with the addition of a 50-fold molar excess of unlabeled IP-001 ligand and imaged at 4 and 24 h. In a parallel independent experiment, three mice belonging to both groups were sacrificed at 4 and 24 h post injection, and their main organs were explanted for measuring the biodistribution of the radiotracer. As per SPECT/CT imaging analysis, the tumor could already be visualized after 4 h, even though [^111^]In-IP-001 showed a high accumulation in all the main organs. Blood radioactivity was high, suggesting that a significant amount of the radiotracer was in circulation or bound to blood constituents, such as serum albumin, as found in the in vitro experiments. The kinetics of clearance appeared to be slow with a tumor to background ratio improving over time up to 24 h, when radioactivity accumulation mainly persisted in the tumor, the gastro-intestinal tract, and the kidneys. Paradigmatic SPECT/CT images are reported in Figure 3. In the biodistribution studies, the tumor achieved the maximal uptake (2.36 ± 0.26% IA/g; *n* = 3) at 4 h post injection despite the greater (blood: 4.21 ± 0.40% IA/g; liver: 8.25 ± 2.21%; kidneys: 6.99 ± 0.97%; spleen: 3.88± 0.36%; *n* = 3) or at least comparable (intestines: 2.39 ± 0.28%; lungs: 2.82 ± 0.21%; pancreas: 2.03 ± 0.16%; *n* = 3) accumulation in other organs and tissues, suggesting a low specific biodistribution of the radiotracer at this time. At 24 h post injection, tumor uptake decreased to 1.52 ± 0.71% IA/g (*n* = 3), but the tumor to organ ratios highly improved with respect to 4 h, as shown in Figure 4. Actually, only the liver and kidneys still exhibited higher uptake as compared to the tumor tissue, with ratios of 0.47 and 0.48, respectively. Conversely, the tumor to muscle (T/M) and tumor to blood (T/B) ratios were highly favorable (15.72 and 10.19, respectively), thus confirming the rapid accumulation and persistence in the tumor visualized by SPECT/CT images, as well as a slow kinetics of clearance that occurred via both renal and hepatobiliary excretion. Residual radioactivity in all the other main organs and, in particular, the intestines and pancreas was low (0.55 ± 0.29% IA/g and 0.26 ± 0.04% IA/g, respectively).

**Figure 5:**
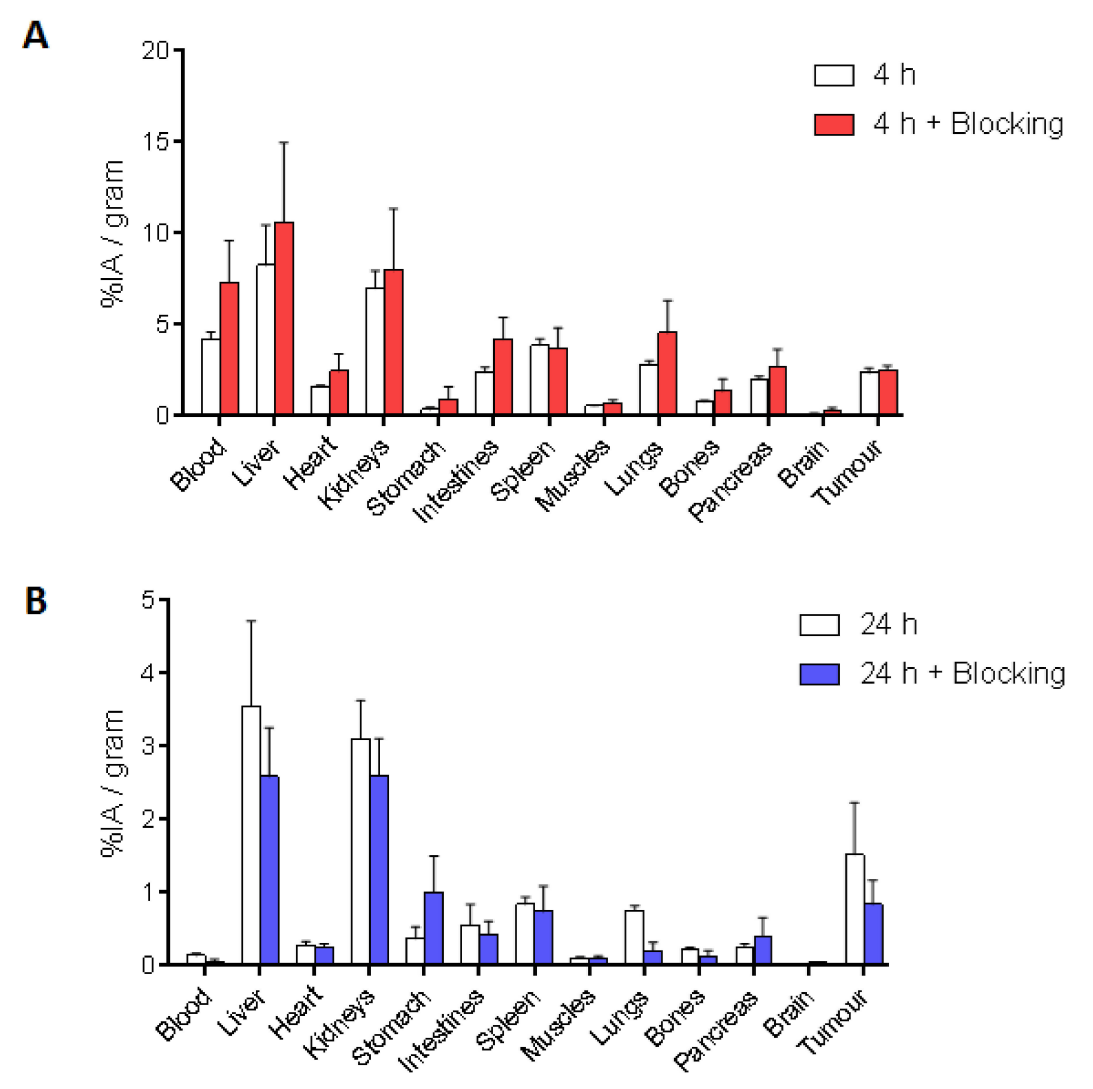
Comparison among the biodistributions in multiple organs of [^111^In]In-IP-001 and [^111^In]In-IP-001, as well as unlabeled-IP-001 (blocking) in A549 cancer-bearing nude mice (*n* = 3; mean ± SD) at 4 (**A**) and 24 h (**B**) post injection.

**Figure 6:**
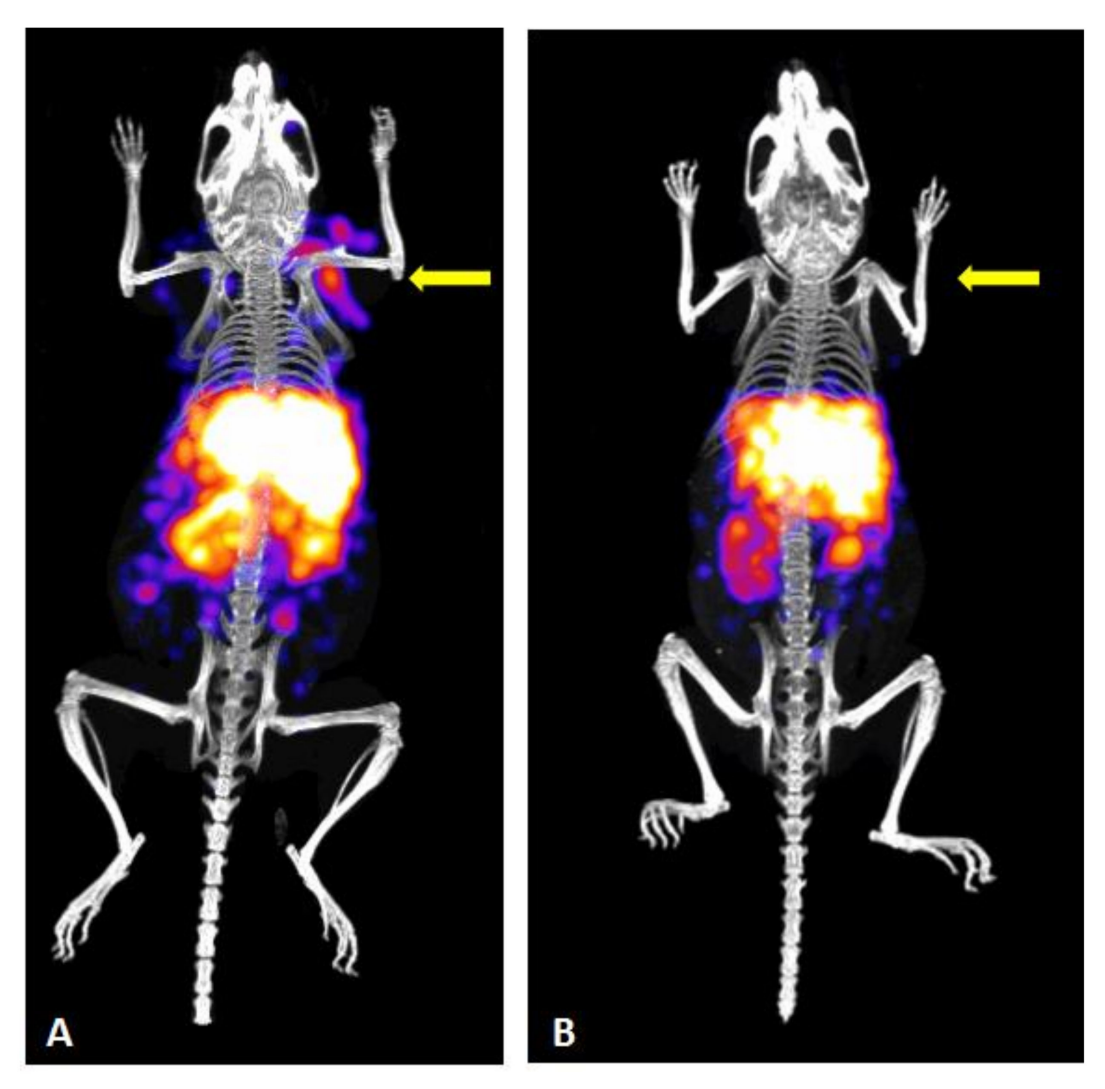
Representative SPECT/CT scans as MIP views of two A549 tumor-bearing mice injected with [^111^In]-IP-001 (**A**), as well as [^111^In]-IP-001 and 50-fold molar excess of unlabeled IP-001 (blocking) (**B**) at 24 h post injection. Mice developing tumors approximately of the same size (about 112 mm^3^) are shown. Tumor position in the shoulder is indicated by the arrow.

At 4 h post injection, a blocking experiment reflected a low specific distribution of the radiotracer, as no significant differences were found in the radioactivity measured in the organs and tumors of the normal and blocked groups. After 24 h, a more pronounced gap could be appreciated when, for instance, the tumor uptake was 1.52 ± 0.71% IA/g (*n* = 3) in the experimental group versus 0.84 ± 0.33% IA/g (*n* = 3) in the blocked group. However, the differences were still not statistically significant (p = 0.08). Complete comparisons between the two groups at 4 and 24 h are shown in Figure 5. On the other hand, a clear effect of the blocking could be visualized in the SPECT/CT images at 24 h post injection. In Figure 6, for instance, it is shown that a negligible uptake in the tumor was found in the blocked mouse in comparison with the unblocked one.

## 3. Discussion

In the present study, a nastorazepide derivative functionalized with a DOTA chelator was synthesized to provide a potential precursor for the diagnosis and systemic therapy of CCK-2R-expressing tumors. In fact, the presence of a chelator moiety allowed for the coordination of several metal radionuclides, thus triggering the possibility of a radiotheragnostic approach. Nastorazepide is a well-known 1,5-benzodiazepine derivative that acts as an antagonist ligand for CCK-2R, and it is currently under development as a therapeutic drug for pancreatic cancer, gastroesophageal reflux disease, and peptic ulcers. The state of the art in the field of imaging and treatment of CCK-2R-expressing tumors with radiopharmaceuticals is mainly focused on the labelling of the natural ligands of this receptor (i.e., CCK and gastrin) or those derived thereof. However, issues regarding in vivo stability, receptors affinity, and high kidney retention have hindered this approach so far. An alternative and less explored pathway is the labelling of small molecules such as 1,5-benzodiazepine derivatives, although, to the best of our knowledge, this has only been pursued with the diagnostic radionuclide technetium-99 and, despite promising preliminary results, only minor efforts have been devoted to this approach [16,17].

**Figure 7:**
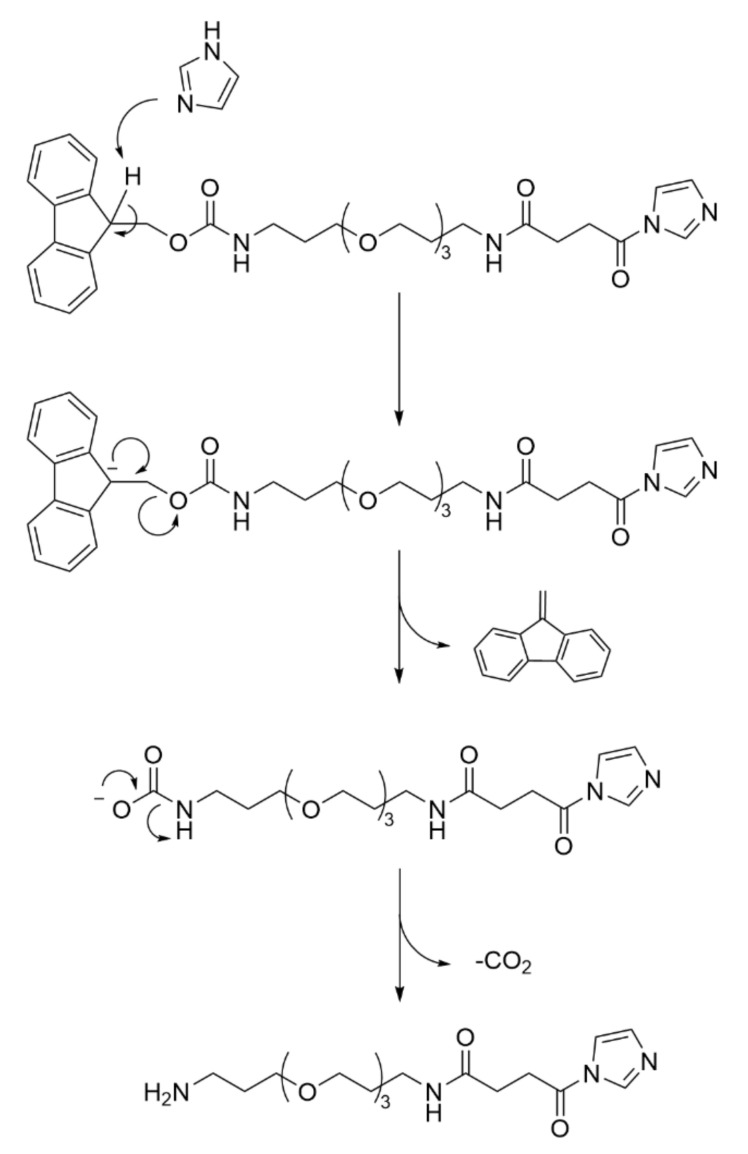
Elimination of the fluorenylmethoxycarbonyl (Fmoc) group from CDI-activated compound **2** mediated by imidazole.

**Figure 8:**
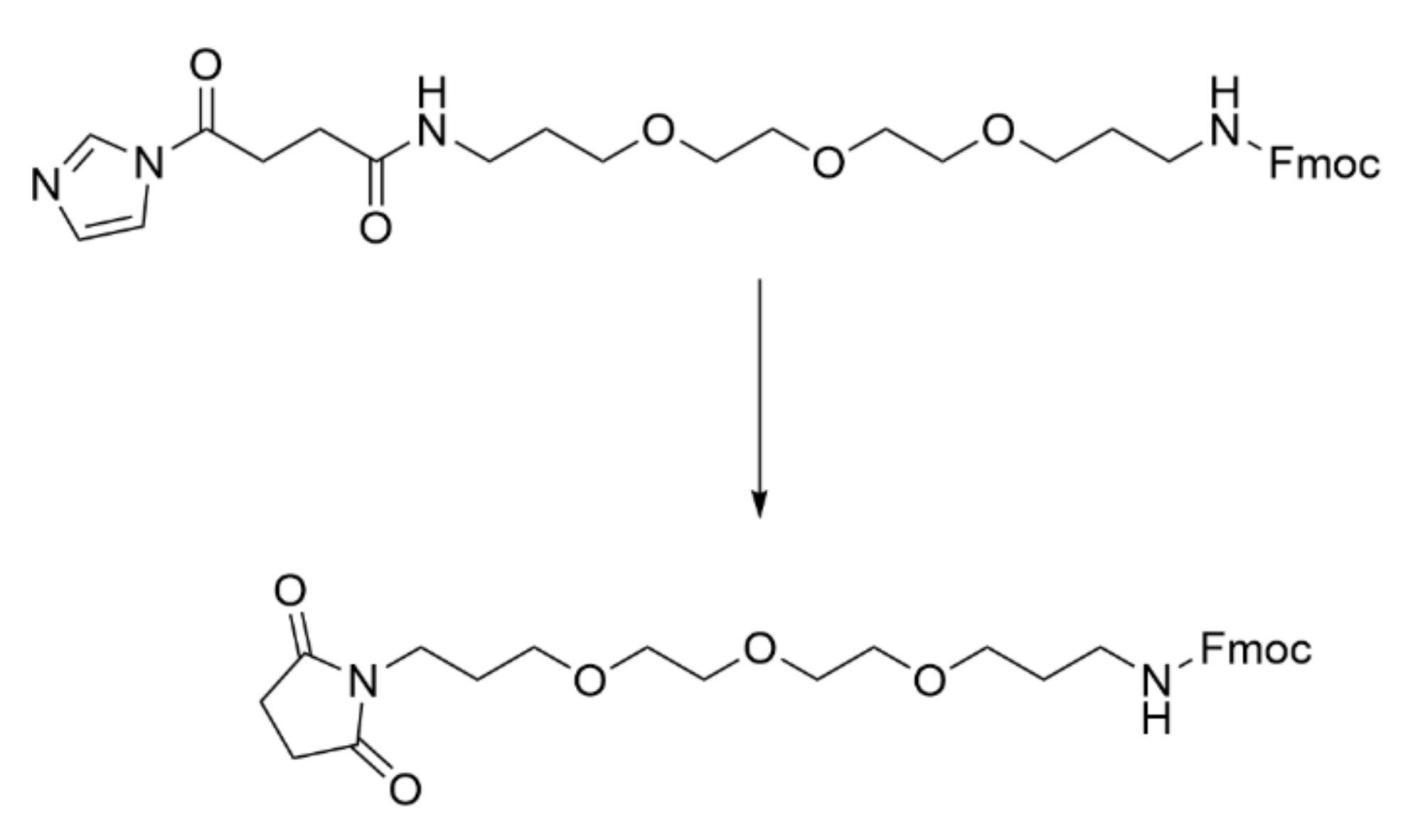
By-product obtained from the cyclization of the CDI-activated succinyl group of compound **2**.

The precursor synthesized here is composed by a nastorazepide moiety connected to a DOTA chelator with a short alkyl chain followed by a PEGylated portion introduced to increase the hydrophilicity of the whole molecule. The linker is an important part of the molecules because it ensures a sufficient separation between the pharmacophore and the radiometal binding moiety, which also influences the affinity. Differently from previously reported works, a linker completely not based on an amino acid sequence was assembled to avoid any possible site of enzymatic cleavage. While a nastorazepide core was synthesized in a six-step reaction pathway, as reported in the literature, the linker was obtained by a condensation reaction between *N*-Boc-1,6-hexanediamine (**1**) and CDI-activated *N^19^*-Fmoc-4-oxo-9,12,15-trioxa-5,19-diazanonadecanoic acid (**2**). Consequently, the Boc protecting group was eliminated, and the linker was again condensed to the CDI-activated nastorazepide core. Finally, the Fmoc moiety was cleaved, and the chelator was inserted. The CDI-activation of the succinyl moiety of compound **2** and, more generally, the activation of the carboxylic groups with CDI in the presence of an Fmoc protecting group (such as reaction steps a and c in Scheme 1) were the trickiest steps of the pathways, yielding 46% and 32%, respectively. The moderate yields were supposed to derive from two side-reactions due to the well-known mechanism of action of CDI that leads to the release of an imidazole group [20]. This group, imparting a basic character to the reaction mixture, could have been responsible for a first side-reaction with compounds **2** and **4** that led to the elimination of the Fmoc-protecting group with the consequent formation of a free amine. The suggested mechanism of this reaction occurring for the CDI-activated compound **2** is reported in Figure 7. Moreover, a second imidazole-mediated side-reaction was supposed to involve compound **2** during the first condensation step of the pathway, thus yielding the cyclization of the succinyl group instead of its activation, as reported in Figure 8. The formation of this compound was confirmed by ^1^H-NMR (Figure S10). To minimize the formation of these by-products, the original work-up or the reactions was modified by adding a 1 M acetic acid solution to the reaction mixtures to fix the pH to around 5 (thus protonating the imidazole and reducing its catalytic behavior) before the evaporation step.

Another step of the synthesis worth attention is the passage from compound **6** to compound **7**, i.e., the elimination of the Fmoc group after the condensation of the linker with Z-360. Conventional elimination conditions suggest the use of a 20% piperidine/DMF solution [21], but, in our case, a very low yield was obtained, probably due to the instability of the linker in a basic environment, as reported for similar chemical structures [22]. For this reason, the use of a milder base, such as a 50% morpholine/DMF solution, was preferred to obtain almost quantitative results [23]. Finally, in an alternative synthetic approach, CDI-activated Z-360 (**5**) was firstly reacted with *N*-Boc-1,6-hexanediamine (**1**), and, after the elimination of the Boc group, the obtained intermediate was further reacted with CDI-activated *N^19^-*Fmoc-4-oxo-9,12,15-trioxa-5,19-diaza-onadecanoic acid (**2**). However, this method did not show significant advantages with respect to the former one, exhibiting the same side-reactions described above and, moreover, implying an elevate loss of Z-360 due to the low yield of the first reaction. For these reasons, it was not pursued further. The overall yield of the process to obtain IP-001 was around 5% (22% starting from compound **5**), which was in line with a multi-step synthetic pathway, with the yield-limiting step being the synthesis of compound **5**. In view of further optimization, attention should be paid to the synthesis optimization of the latter.

The radiolabeling of IP-001 with indium-111 was performed in the standard conditions involving the labelling of a DOTA-conjugated small molecule with trivalent radiometals [24], obtaining an incorporation higher than 95% in a quite straightforward way. Stability in all tested conditions, and particularly in human serum and blood, was high and generally comparable at short incubation times (<24 h) to the best peptide-based candidates for the treatment of CCK-2R-expressing tumors, but it was much superior at longer times (intact amount of [^111^In]In-IP-001 >93% after 120 h in HB). On the other hand, the amount of [^111^In]In-IP-001 bound to the serum protein was almost comparable to other indium-111-labelled minigastrin analogues [13]. A direct comparison with other non-peptide-based radiotracers was not possible since, to the best of our knowledge, the high stability in HB at a late time found here was the first reported experimental confirmation of previous theoretic assumptions for this kind of molecular structure.

CCK-2R expression has been reported in cancers of different origins by immunohistochemistry on tumor specimens [25]. However, CCK-2R expression in human cancer cell lines has largely not been investigated. CCK-2R expression at the mRNA level has been reported in human PC3 prostate carcinoma, U373 glioma, U2OS osteosarcoma, Colo205 colon carcinoma [26], HepG2 hepatoma [27], gastric and colorectal cell lines, and MCF-7 breast cancer and Molt4 lymphoblastic leukemia cell lines [28]. To date, studies on CCK-2R-targeting probes for cancer imaging have only been performed by exploiting transfected cell lines (the human A431 cancer cell line, HEK293 human embryonic kidney cells, NIH 3T3 mouse embryonic fibroblast cells, and Chinese hamster ovary cells) or rat pancreatic tumor cells [16,24,29,30,31,32,33]. However, the demonstration of CCK-2R overexpression by transfected versus parental cells has generally been lacking.

Herein, we demonstrated that the CCK-2R protein is indeed expressed by various human cancer cell lines, and we decided to perform experiments in vitro and in vivo on A549 cells as representative (Figure 1). We acknowledge the fact that our results are only partially comparable to other studies reported thus far, but we would like to emphasize that non-transfected tumor cells represent a closer model to the real physio-pathological condition than transfected cancer cell lines. With this in mind, we ascertained that [^111^In]In-IP-001 cell uptake in vitro was two-fold lower than what reported previously in AR4-2J rat pancreatic tumor cells for indium-111-labelled minigastrin derivatives [24] and four-fold lower than the total amount of technectium-99m nastorazepide-based derivatives accumulated by CCK-2R-transfected HEK293 cells [17].

In BALB/c nude mice developing A549 cell-derived tumors, [^111^In]In-IP-001 exhibited a quite rapid absorption rate in all the main organs (liver: 8.25 ± 2.21%; kidneys: 6.99 ± 0.97%; spleen: 3.88 ± 0.36%) and the tumor (2.36 ± 0.26% IA/g) within 4 h. A high amount of injected radioactivity was also found in the circulation, suggesting a strong interaction with serum albumin (Figure 3 and Figure 4). Clearance was slow and performed by both the renal and hepatobiliary pathways, probably due to the relatively high lipophilicity of the radiotracer. After 24 h, the kidneys and liver (3.11 ± 0.52% IA/g and 3.55 ± 1.17% IA/g, respectively) remained the organs with the highest uptake, followed by the tumor (1.52 ± 0.71% IA/g), which was clearly delineated in the CT/SPECT images (Figure 3). Moreover, at 4 h post-injection, a certain level of radioactivity was also detected in the intestines, lungs, and pancreas (no statistically significant difference with the tumor), with a decrease of the signal of more than 73% over 24 h, indicating a transient accumulation. On the contrary, the tumor tissue still retained 65% of the accumulated [^111^In]In-IP-001 at 24 h post-injection. The lungs, pancreas, and intestines exhibited a more rapid wash-out with tumor/organ ratios of 2.02, 5.85, and 2.76, respectively (Figure 4). The slow kinetics of radiotracers based on the nastorazepide core was already reported, although a significantly more promising biodistribution was obtained in mice bearing tumors derived from transfected HEK293 cells [16,17], in which kidneys were the only organ with a comparable uptake with respect to the tumor at delayed times. As reported in Table 1, the addition of a PEGylated spacer and a strongly hydrophilic chelator like DOTA to the hydrophobic Z-360 core imparted a quite hydrophilic nature to IP-001. Indeed, at pH = 7.2, the chelator was completely dissociated, and the precursor was in the anionic form (i.e., IP-001^3−^). However, it is reasonable that the formation of a neutral complex with [^111^In]In^3+^ increased the lipophilicity of the final radiotracer (experimental value of 0.45), thus giving reason of its unspecific accumulation in the liver and spleen. Hence, further chemical optimizations are required, with a particular focus on the hydrophilicity of the linker, to obtain a novel ligand with a logP remarkably lower than −3.22. In general, [^111^In]In-IP-001 biodistribution reflected the behavior of a radiotracer with a moderate receptor affinity, as attested by the blocking studies performed with an excess of unlabeled ligand in which, despite the results showing a trend of lower [^111^In]In-IP-001 accumulation in the blocking setting (45% lower uptake in the tumor tissue), no statistically significant differences between the two groups of injected mice were found. Definitely, although a high stability was achieved by avoiding the use of peptides or peptide-mimetic moieties on the linker, affinity and hydrophilicity could likely be further tailored and enhanced by acting on this portion of the structure.

## 4. Materials and Methods

### 4.1. General Procedures and Chemicals

All chemicals (Acros, Aldrich, IrisBiotech suppliers, Marktredwitz, Germany) were reagent grade and used without further purification unless otherwise specified. Solvents (Carlo Erba, Milan, Italy and Lab Scan, Bangkok, Thailand) were obtained as analytical grade and degassed by ultra-sonication for 15–20 min before use. Deuterated solvents (SG Isotec, Pančevo, Republic of Serbia) used for NMR analysis exhibited an isotopic purity of 99.5%. pH measurements were conducted using a calibrated pH-meter (Mettler-Toledo, Biosigma, Venice, Italy). All reaction intermediates were purified as specified in the following procedures and their purity was ≥95% by NMR and/or HRMS. Indium-111 chloride solutions (370 MBq in 0.5 mL) were purchased from Curium (Milan, Italy).

### 4.2. Instrumentations

NMR spectra were recorded by means of a Bruker 400-AMX spectrometers using tetramethylsilane (TMS) as the internal standard. For each sample, 1–2 mg were weighed and diluted up to 0.6 mL with the deuterated solvent described in the specific synthetic paragraph into a 5 mm NMR tube. A 90° pulse was calibrated for each sample, and standard NMR parameters were used.

Mass spectrometry was performed through the direct injection of the sample on an Applied Biosystem Mariner System 5220 equipped with a MALDI TOF/TOF 400 Plus AB Sciex, (Framingham, MA, USA) analyzer.

The purification of the precursor (IP-001) was carried out by an Agilent 1290 Infinity II preparative HPLC equipped with a binary pump and UV–Visible detector. A semi-preparative Zobrax-Eclipse (Agilent) (C18; 5µm; 250 × 21.2 mm) column was used. Eluents were H_2_O + 0.1% TFA (A) and ACN (B) at flow = 17mL/min with the following gradient: minutes 0–2 A: 90% B: 10%; minutes 2–23 B: 10% -> 76%; minutes 23–27 B: 76% -> 100%; and minutes 27–28 B: 100% -> 10%. The detection of reaction products was performed, evaluating absorbance at λ = 225 nm. During the purification process, IP-001 showed a retention time of 19 min.

HPLC analyses on indium-111-labelled IP-001 were performed on an S1125 HPLC pump system, and the column used was an Acclaim 120 C18, 3 × 150 mm, 3 µm 120 Å pore size; flow: 0.6 mL/min; gradient: minutes 0–2 A: 70% B: 30%; minutes 2–12 B: 30% -> 95%; and minutes 12–15 B: 95% -> 30%, where A: H_2_O (0.1% TFA) and B: MeCN. Detection was carried out with an S3245 UV–Vis and an S3700 gamma detector system.

TLC plates used for the assessment of the preparative reactions were silica gel 60 F245 (0.2 mm, Merck) with the eluents mentioned for the specific product in the following paragraphs. Column chromatography was carried out with silica gel 60 (0.063–0.100 mm, Merck), and elution was performed using the eluent described for the specific preparations. The radioactive incorporation and stability of [^111^In]In-IP-001 were assessed on ITLC-SG plates, developed in a 1 M ammonium acetate/MeOH 1:1 *v*/*v* solution, by using a flatbed-imaging scanner (Cyclone, Perkin Elmer).

### 4.3. Synthesis of N^1^-Boc-N^25^-Fmoc-8,11-dioxo-16,19,22-trioxa-7,12-diazapentacosane-1,25-diamine (3)

**Figure 9:**
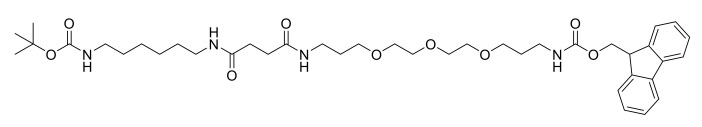
Chemical structure of **3**.

CDI (0.2 g, 1.5 mmol) was added to a 32 mL MeCN solution of N^19^-Fmoc-4-oxo-9,12,15-trioxa-5,19-diazanonadecanoic acid (**2**; 0.5 g, 1.0 mmol), and the mixture was stirred and heated to reflux for 1 h (TLC: CHCl_3_/MeOH 9:1). After cooling, triethylamine (TEA) (0.2 g, 1.5 mmol) was added to the mixture, followed, after 10 min, by a solution of N-Boc-1,6-hexanediamine (**1**; 0.3 g, 1.2 mmol) in MeCN (20 mL; dropwise addition). The solution was stirred at room temperature for 2 h (TLC: CHCl_3_/MeOH 9:1). The mixture was than acidified to pH 5 with a 1 M CH_3_COOH solution in MeCN and concentrated to dryness. The residue was dissolved in 40 mL of ethyl acetate (EtOAc) and washed with a saturated NH_4_Cl solution (20 mL × 2). The organic phase was then concentrated to dryness, and the product was purified by column chromatography (eluent: CHCl_3_/MeOH 95:5) to yield **3** (0.3 g, 0.46 mmol, 46% yield, chemical structure in Figure 9).

^1^H-NMR (400 MHz, CDCl_3-_d)δ ppm 7.75 (d, *J* = 7.6, 2H); 7.60 (d, *J* = 7.6, 2H); 7.39 (t, *J* = 7.6, 2H); 7.30 (t, *J* = 7.6, 2H); 6.66 (broad s, 1H); 6.40 (broad s, 1H); 5.67–5.59 (m, 1H); 4.62 (broad s, 1H); 4.45–4.35 (m, 2H); 4.24–4.17 (m, 1H); 3.65–3.49 (m, 12H); 3.35–3.25 (m, 4H); 3.22–3.14 (m, 2H); 3.12–3.02 (m, 2H); 2.46 (s, 4H); 1.83–1.68 (m, 4H); 1.50–1.44 (m, 4H); 1.42 (s, 9H); 1.34–1.26 (m, 4H).

### 4.4. Synthesis of N^25^-Fmoc-8,11-dioxo-16,19,22-trioxa-7,12-diazapentacosane-1,25-diamine (4)

**Figure 10:**
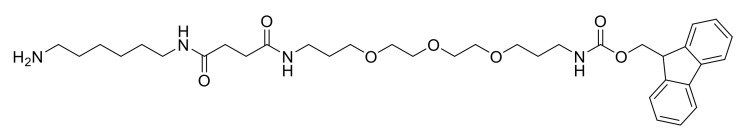
Chemical structure of **4**.

A solution of **3** (0.3 g, 0.5 mmol) in DCM (3.5 mL) was cooled to 0 °C by an ice bath for 20 min, and then TFA (1 mL) was added dropwise. The mixture was stirred for 1 h at room temperature (TLC: CHCl_3_/MeOH 9:1) and then evaporated to dryness to obtain **4** (0.2 g, 0.5 mmol, quantitative yield, chemical structure in Figure 10).

^1^H-NMR (400 MHz, CDCl_3-_d)δ ppm 7.76 (d, *J* = 7.6, 2H); 7.58 (d, *J* = 7.6, 2H); 7.40 (t, *J* = 7.6, 2H); 7.31 (t, *J* = 7.6, 2H); 4.56–4.36 (m, 2H); 4.26–4.16 (m, 1H); 3.66–3.44 (m, 12H); 3.36–3.16 (m, 6H); 3.08–2.94 (m, 2H); 2.63 (s, 4H); 1.82–1.60 (m, 6H); 1.54–1.24 (m, 6H).

### 4.5. Synthesis of N^1^-{3-[3-(1-pivaloylmethyl-5-cyclohexyl-2,3,4,5-tetrahydro-1H-benzo[b][1,4]diazepin-2-one-3-yl)]ureido}benzoyl-N^25^-Fmoc-8,11-dioxo-16,19,22-trioxa-7,12-diazapentacosane-1,25-diamine (6)

**Figure 11:**
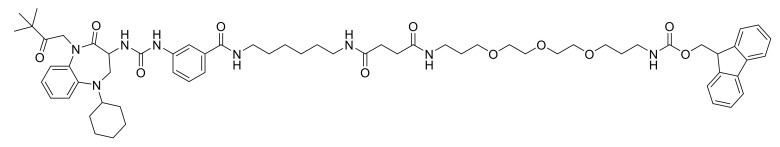
Chemical structure of **6**.

CDI (68.0 mg, 0.4 mmol) was added to a 10 mL MeCN suspension of Z-360 (**5**; 0.2 g, 0.3 mmol), and the mixture was stirred and refluxed for 1 h (TLC: CHCl_3_/MeOH 95:5). After cooling, TEA (42 mg, 0.4 mmol) was added dropwise to the mixture, followed, after 10 min, by a solution of **4** (0.2 g, 0.3 mmol) in MeCN (10 mL; dropwise addition). The solution was stirred at room temperature for 1 h (TLC: CHCl_3_/MeOH 9:1). The mixture was than acidified to pH 5 with a 1 M CH_3_COOH solution in MeCN and concentrated to dryness. The residue was dissolved in 40 mL of EtOAc and washed with a saturated NH_4_Cl solution (20 mL × 2). The organic phase was then concentrated to dryness, and the product was purified by column chromatography (eluent: EtOAc/EtOH 95:5, followed byCHCl_3_/MeOH 95:5) to yield **6** (0.1 g, 0.1 mmol, 32% yield, chemical structure in Figure 11).

HRMS *m*/*z* = 1143.6494 [M+H^+^] (calculated); 1143.6495 [M+H^+^] (found). ^1^H-NMR (400 MHz, CDCl_3-_d)δ ppm 7.75 (d, *J* = 7.6, 2H); 7.67–7.52 (m, 3H); 7.38 (t, *J* = 7.6, 2H); 7.30 (t, *J* = 7.6, 2H); 7.25–7.19 (m, 2H); 7.17–7.01 (m, 4H); 6.98–6.93 (m, 1H); 5.24 (d, *J* = 17.9, 1H); 4.80–4.78 (m, 1H); 4.46–4.32 (m, 2H); 4.24–4.16 (m, 1H); 4.11 (d, *J* = 17.9, 1H); 3.66–3.10 (m, 23H); 2.54–2.40 (m, 4H); 2.10–2.00 (m, 2H); 1.88–1.26 (m, 20H); 1.15 (s, 9H).

### 4.6. Synthesis of N^1^-{3-[3-(1-pivaloylmethyl-5-cyclohexyl-2,3,4,5-tetrahydro-1H-benzo[b][1,4]diazepin-2-one-3-yl)]ureido}benzoyl-8,11-dioxo-16,19,22-trioxa-7,12-diazapentacosane-1,25-diamine (7)

**Figure 12:**
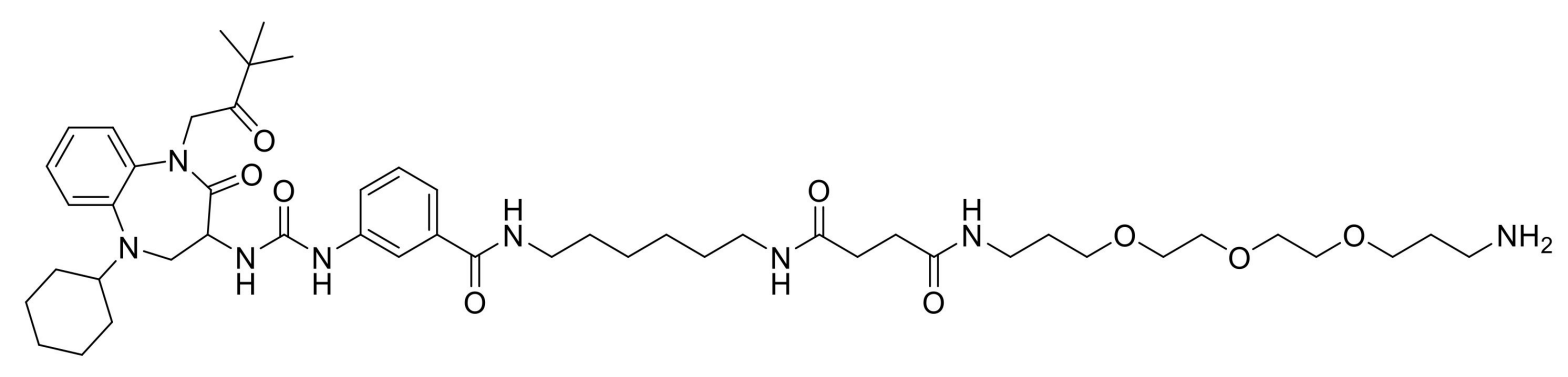
Chemical structure of **7**.

A DMF solution (2 mL) of **6** (0.1 g, 0.1 mmol) was cooled and stirred in an ice bath for 10 min. Then, under constant cooling and stirring, morpholine (2 mL) was added, and the mixture was reacted for 1.5 h at room temperature. The completion of reaction was assessed by TLC (CHCl_3_/MeOH 9:1). The mixture was brought to dryness through an azeotropic distillation with toluene, and the residue was purified by column chromatography (eluent: CHCl_3_/MeOH 9:1) to obtain **7** (0.1 g, 97 µmol, 97% yield, chemical structure in Figure 12).

HRMS *m*/*z* = 921.5814 [M+H^+^] (calculated); 921.5806 [M+H^+^] (found). ^1^H-NMR (400 MHz, MeOD-d_4_)δ ppm 7.76–7.73 (m, 1H); 7.48–7.43 (m, 1H); 7.40–7.35 (m, 1H); 7.34–7.25 (m, 3H); 7.11–7.03 (m, 2H); 5.19 (d, *J* = 17.9, 1H); 4.61–4.53 (d, *J* = 7.0, 1H); 4.40 (d, *J* = 17.9, 1H); 3.65–3.34 (m, 18H); 3.25–3.18 (m, 3H); 3.10–3.05 (m, 2H); 2.45 (s, 4H); 2.12–2.02 (m, 1H); 1.95–1.26 (m, 21H); 1.23 (s, 9H).

### 4.7. Synthesis of N^1^-{3-[3-(1-pivaloylmethyl-5-cyclohexyl-2,3,4,5-tetrahydro-1H-benzo[b][1,4]diazepin-2-one-3-yl)]ureido}benzoyl-N^25^-{2-[4,7,10-tri(tertbutoxycarbonylmethyl)-1,4,7,10-tetrazacyclododecan-1-yl]acetyl}-8,11-dioxo-16,19,22-trioxa-7,12-diazapentacosane-1,25-diamine (8)

**Figure 13:**
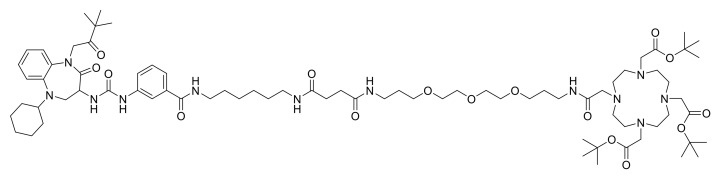
Chemical structure of **8**.

BOP (35.0 mg, 80 μmol) and TEA (16.0 mg, 0.2 mmol) were added to a 2 mL DMF solution of DOTA(tBu)_3_ ester (40.0 mg, 71.0 μmol). The mixture was stirred for 10 min, and then a 2 mL DMF solution of **7** (74.0 mg, 80.0 μmol) was added dropwise. The solution was reacted under stirring for 24 h at room temperature (TLC: CHCl_3_/MeOH 9:1). Then, it was concentrated to dryness, re-dissolved in 10 mL EtOAc, and finally washed with a saturated water solution of NH_4_Cl (5 mL × 2). The organic phase was concentrated to dryness, and the obtained residue was purified by column chromatography (eluent: CHCl_3_/MeOH 9:1) to yield **8** (30.7 mg, 21.2 mmol, 30% yield, chemical structure in Figure 13).

HRMS *m*/*z* = 1475.9493 [M+H^+^] (calculated); 1475.9584 [M+H^+^] (found). ^1^H-NMR (400 MHz, CDCl_3-_*d*)δ ppm 8.07 (broad s, 1H); 7.68–7.56 (m, 2H); 7.40–7.28 (m, 2H); 7.24–7.18 (m, 3H); 6.84–6.78 (m, 1H); 6.60–6.52 (m, 1H); 5.25 (d, *J*=17.9, 1H); 4.78–4.66 (m, 1H); 4.13 (d, *J* = 17.9, 1H); 3.64–3.12 (m, 35H); 2.68–2.42 (m, 16H); 2.10–2.00 (m, 2H); 1.88–1.50 (m, 16H); 1.46–1.42 (m, 27H); 1.37–1.33 (m, 4H); 1.17 (s, 9H).

### 4.8. Synthesis of N^1^-{3-[3-(1-pivaloylmethyl-5-cyclohexyl-2,3,4,5-tetrahydro-1H-benzo[b][1,4]diazepin-2-one-3-yl)]ureido}benzoyl-N^25^-{2-[4,7,10-tri(carboxymethyl)-1,4,7,10-tetrazacyclododecan-1-yl]acetyl}-8,11-dioxo-16,19,22-trioxa-7,12-diazapentacosane-1,25-diamine (IP-001)

**Figure 14:**
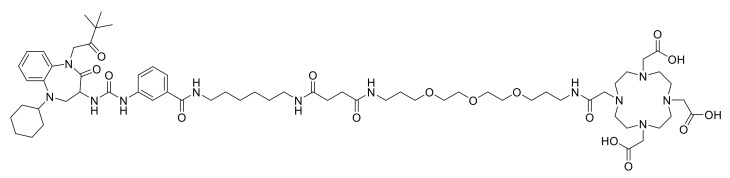
Chemical structure of **IP-001**.

A solution of **8** (30.7 mg, 21.0 μmol) in 2 mL of DCM was pre-cooled to 0 °C for 20 min in an ice bath. Then, TFA (0.6 mL) was added dropwise, and the mixture was stirred overnight at room temperature. After the assessment of the completion of the reaction by TLC (CHCl_3_/MeOH 9:1), the solution was evaporated to dryness, and the product was purified by preparative HPLC. IP-001 was obtained in a quantitative yield (27 mg, 21.0 μmol, purity > 95%, chemical structure in Figure 14). Lipophilicity and other pharmacokinetic properties for the IP-001 and Z-360 were predicted by using the SwissADME web tool, as reported in [34].

HRMS *m*/*z* = 1307.7615 [M+H^+^] (calculated); 1307.7650 [M+H^+^] (found). ^1^H-NMR (400 MHz, MeOD-*d_4_*)δ ppm 7.88 (d, *J* = 8.1, 1H); 7.74 (d, *J* = 8.1, 1H); 7.60–7.53 (m, 1H); 7.52–7.46 (m, 1H); 7.44–7.25 (m, 3H); 7.15–7.02 (m, 1H); 5.20 (d, *J* = 17.9, 1H); 4.60–4.46 (m, 1H); 4.39 (d, *J* = 17.9, 1H); 3.65–3.45 (m, 14H); 3.40–3.15 (m, 16H); 2.46 (s, 4H); 2.12–2.03 (m, 1H); 1.80–160 (m, 4H); 1.64–1.56 (m, 4H); 1.54–1.44 (m, 4H); 1.40–1.30 (m, 10H); 1.24 (s, 9H).

### 4.9. Labelling of IP-001 with Indium-111

A 0.8 mL solution of a 0.05 M HCl and a 60 μL 1.5 M sodium acetate solution (pH 4.66) were placed in a 2 mL microtube. To this mixture, 50 μL of a 1 mg/mL IP-001 water solution was added, followed by 0.2 mL of an indium-111 chloride (60–130 MBq) solution. The reaction was heated at 95 °C for 15 min, and aliquots were analyzed by HPLC and radio-TLC to determine the labelling efficiency. When necessary, the labelling mixture was purified by SPE using a light C-18 cartridge. The cartridge was firstly conditioned with 3 mL of EtOH followed by 3 mL of water. Afterwards, sample was loaded and the cartridge was washed with a hydro-alcoholic solution (1.5 mL, water/EtOH 80:20) to remove unlabeled [^111^In]In^3+^. The radiotracer ^111^[In]In-IP-001 was then eluted with EtOH (0.4 mL) and diluted with water (2 mL). All experiments were performed in triplicate.

### 4.10. Stability, Serum Proteins Binding and Lipophilicity Studies

The stability of [^111^In]In-IP-001 solutions (0.5 mL, 10 nmol, 18 MBq) was assessed by means of radio-TLC or RP-HPLC at various temperatures (4, 24, and 37 °C) and in the presence of different media up to 120 h after preparation. The studies were performed by incubating the radiotracer with (i) a 0.9% NaCl solution at a 9:1 ratio, (ii) a 0.1 M HEPES solution at a 9:1 ratio, (iii) a 0.1 M EDTA solution at 9:1 and 1:1 ratios, (iv) human serum (HS) at a 9:1 ratio, and (v) HB at a 1:1 ratio. After TLC analysis, samples incubated with HB were centrifuged at 3000 rpm for 10 min to precipitate blood cells, and 200 μL of a MeCN/H_2_O/TFA 50/45/5 *v*/*v*/*v* solution was added to 400 μL of the supernatant. After another centrifugation under the same conditions, the supernatant was discarded and the residual radioactivity due to the proteins bound fraction was measured in a dose calibrator. Lipophilicity calculation were performed in octanol–water with the shaking flask method, as already described elsewhere [13]. All experiments were performed in triplicate.

### 4.11. Selection of the Human Cell Line

#### 4.11.1. Cell Cultures

A series of human cancer cell lines (A549, A431, PC3, MCF-7, SK-BR-3, MDA-MB-23, HT-29 and HCC-2998) were screened regarding the expression of CCK-2R. The A549 non-small cell lung cancer and A431 skin cancer cell lines were purchased from the European Collection of Authenticated Cell Cultures (ECACC 86012804 and ECACC 85090402). The PC3 prostate cancer cell line; the MCF-7, SK-BR-3, and MDA-MB-231 breast cancer cell lines; and the HT-29 and HCC-2998 colon cancer cell lines were kindly provided by Dr. Alessia Ciarrocchi (Laboratory of Translational Research AUSL-IRCCS, Reggio Emilia, Italy). A549, A431, PC3, MCF-7, and HCC-2998 were cultured in RPMI-1640 added of 10% heat-inactivated FBS. SK-BR-3, MDA-MB-231, and HT-29 were cultured in DMEM with high glucose added to 10% heat-inactivated FBS. Media contained 100 U/mL penicillin-streptomycin and glutaMAX. Cell culture reagents were purchased from Thermo Fisher (Milan, Italy). Cell lines were routinely maintained at 37 °C with 5% CO_2_.

#### 4.11.2. Screening of the Cell Lines by Western Blot

To avoid the use of trypsin, cells were lysed in situ with a 500 μL RIPA lysis buffer supplemented with protease-inhibitors (RIPA lysis buffer system, sc-24948, Santa Cruz Biotechnologies) at 4 °C for 30 min. Lysates were clarified by centrifugation at 12,000× *g* at 4 °C for 15 min, and then protein concentrations were measured using the DC Protein Assay (Bio-Rad). About 40 µg of proteins were separated using Bolt Bis-Tris Plus precast 8% polyacrylamide gels with an MOPS SDS running buffer (Thermo Fisher) then blotted onto PVDF membranes. After blocking with TBS containing 0.1% Tween 20 and 5% BSA for 2 h at room temperature, and membranes were incubated overnight at 4 °C with (anti-human CCK-2R) mouse monoclonal antibodies conjugated with phycoerythrin (clone E-3, sc-166690, Santa Cruz Biotechnology) diluted 1:400 in TBS containing 0.1% Tween 20 and 5% BSA. Fluorescent signals were detected with the ChemiDoc instrument (Bio-Rad) using a Cy3 filter. To check the protein load, membranes were afterwards incubated overnight at 4 °C with rabbit anti-human GAPDH antibodies (Santa Cruz Biotechnologies) diluted 1:500 in TBS containing 0.1% Tween 20 and 5% BSA. Signals were detected by 1-h incubation at room temperature with HRP-conjugated secondary antibodies (Abcam, AB6013), followed by incubation with an ECL detection reagent (Thermo Fisher) and ChemiDoc imaging (Bio-Rad).

#### 4.11.3. Confirmation of CCK-2R Membrane Expression by Flow Cytometry

Cells of the selected line (A549) were detached with 5 mM EDTA then stained with 100 µL of phosphate buffer saline (PBS) containing 0.1% Live–Dead Fixable Dead Cell Stain near-IR-fluorescent reactive dye (Molecular Probes) on ice for 30 min to exclude dead cells from the analysis. After washing with PBS added to 1% heat-inactivated FBS (PBS/FBS), cells were incubated on ice for 30 min with 50 μL of PBS/FBS containing 20 μg/mL of anti-CCK-2R-phycoerythrin antibodies (clone E-3, sc-166690, Santa Cruz Biotechnology), 40 μg/mL of anti-CCK-2R-Dy488 antibodies (LS-C756504, LSBio), or 40 μg/mL of CCK-8 peptide (Asp-Tyr-Met-Gly-Trp-Met-Asp-Phe) labelled with FAM supplied by GenScript Biotech (Piscataway, New Jersey, USA). Isotype control antibodies labelled with phycoerythrin (20 μg/mL, Santa Cruz Biotechnology) were used to check non-specific binding. After washing with PBS/FBS, cells were analyzed with the FACSCantoII flow cytometer (BD).

### 4.12. [111. In]In-IP-001 Cellular Uptake

A549 epithelial lung cancer cells were seeded in 6-well plates (20,000/cm^2^) and allowed to adhere overnight. The following day, the medium was removed and radiotracer uptake was studied by incubating 2 × 10^5^ cells at 37 °C in 2 mL of culture medium added to 30 µL (ca. 2 MBq, 1.35 μg, 1 nmol) of [^111^In]In-IP-001. Uptake was monitored at 2, 4, 8, and 24 h. At these time points, the medium was removed and cells were washed twice with 2 mL of ice-cold PBS. Finally, cells were detached with 2 mL of a 0.25% trypsin/EDTA solution at 37 °C and centrifuged to separate the supernatant from the cells pellet. The radioactivity associated with the pellets was measured in a γ-spectrometer and corrected for decay. All experiments were performed in triplicate.

### 4.13. Animal Hosting, Inoculation and Monitoring

Homozygous female BALB/c nude J:NU (JAX stock number 007850) mice were purchased from Jackson Laboratories (Bar Harbor, ME, USA). Mice were 4 weeks old at arrival, and they were hosted in an IVC system (Smart Flow AHU, Tecniplast) with autoclaved rodent bedding hosting 3–4 mice per cage. Mice were fed with autoclaved rodent food and water ad libitum. The inoculation of A549 tumor cell line was performed by the subcutaneous injection of 5 × 10^6^ cells/mouse in 100 uL of fetal bovine serum-free medium (RPMI added of 10% FBS, 1% penicillin-streptomycin, 1% glutamine) into the right shoulder. The cell line was found to be free of mycoplasma contamination, as visually judged by microscopic inspection and by regular 4′,6-diamidino-2-phenylindole (DAPI) staining of the cell cultures. Inoculation was carried out at 5 weeks of age after one week in the facility for acclimatization purposes. Mice were monitored every 2–3 days by measuring weight and tumor dimensions with an electronic caliper. For this purpose, two perpendicular dimensions were noted and volumes of the tumors were calculated with the formula 0.5 × L × W^2^, where L is the measurement of the longest axis and W is the measurement of the axis perpendicular to L, in millimeters [16].

### 4.14. Animal Injection and Imaging

Tumors were allowed to grow for about five weeks until they reached 0.5–1.0 cm of size in one dimension [35,36] (i.e., around 50–250 mm^3^ volume), and then mice were divided in two groups. The experimental group (*n* = 5) was intravenously injected (retro-orbital venous sinus) [37,38] with 150 μL of a [^111^]In-IP-001 solution (2.06 nmol, 7.4MBq), while the blocked group (*n* = 5) was injected with 150 μL of a [^111^]In-IP-001 solution added to a 50-fold molar excess of cold ligand. All injections were performed with the animals under isoflurane anesthesia (1–3%), using 30G ultra-fine insulin syringes. Real-time, live, and fast dynamic screening studies were performed right after injection and on selected time points post-injection, on a dedicated desktop, mouse-sized, planar scintigraphic system (γ-eye^TM^ by BIOEMTECH, Athens, Greece). The system supports fusion with a digital mouse photograph for anatomical co-registration. The main detector is based on two position-sensitive photomultiplier tubes coupled to a CsI(Na) pixelated scintillator and a medium-energy lead collimator with parallel hexagonal holes that supports a range of SPECT isotopes. The system’s field of view is 5 × 10 cm^2^, with a spatial resolution of ~2 mm. For the planar imaging, mice were kept under isoflurane anesthesia and under a constant temperature of 37 °C, and scans had a duration of 10 min. This allowed for a fast test, right after injection, to check whether the injection was successful, and on later time points to quickly evaluate the bio-kinetics of the tracer. Tomographic SPECT/CT imaging was performed with y-CUBE^TM^ and x-CUBE^TM^ (Molecubes, Belgium), respectively, at the same selected time points post-injection. The SPECT system provides a spatial resolution of 0.6 mm for mouse imaging and of 1.5 mm for rat imaging. The CT system performs a spiral scan, it can provide images with 100 um resolution, and images were acquired with 50 kVp. Mouse imaging was performed by keeping the mice anaesthetized under isoflurane and under a constant temperature of 37 °C. SPECT scans were acquired with a 45 min duration, and each SPECT scan was followed by a high-resolution CT scan for co-registration purposes. The SPECT data were reconstructed through a maximum-likelihood expectation-maximization (MLEM) algorithm, with a 500 μm voxel size and 100 μm iterations. CT data were reconstructed through an iterative image space reconstruction algorithm (ISRA) algorithm with a 100 μm voxel size. Tomographic images are presented through maximum intensity projection (MIP) view). The experimental group was imaged at 2, 4, 8, and 24 h post-injection with both imaging systems, while the blocked group was imaged at 4 and 24 h.

### 4.15. Ex Vivo Biodistribution

For biodistribution studies, the injections were performed as described above, and 3 mice for both groups were sacrificed at 4 and 24 h post injection using 2% isoflurane. Main organs and tissues were removed, weighed, and counted together with blood samples, muscle, and urine by a γ-counter system (Cobra II from Packard, Canberra, ME, USA). Results were expressed as a mean percentage ± SD of the injected activity per g (%IA/g) per organ or tissue. For total blood radioactivity calculation, blood was assumed to be 7% of the total body weight.

### 4.16. Ethical Approval

The protocol described and all the animal procedures were approved by the General Directorate of Veterinary Services (Athens, Attica Prefecture, Greece) and by the Bioethical Committee of BIOEMTECH Laboratories (Permit number: EL 25 BIOexp 045) on the basis of the European Directive 2010/63/EU on the protection of animals used for experimental purposes.

### 4.17. Statistical Analysis.

Student’s *t*-test was used to determine whether there were any statistically significant differences between the means of two independent (unrelated) groups. The threshold for statistical significance was set at *p* < 0.05.

## 5. Conclusions

In this study, a CCK-2R-targeting ligand based on the nastorazepide core was synthetized and functionalized with a DOTA chelator with the aim to provide a suitable platform for a theragnostic approach with radioactive metals. Avoiding the use of peptide-based sequences in the structure, including the linker, allowed us to obtain a molecule with high stability in physiological media and that could be easily labelled with indium-111 as pivotal radionuclide for future studies. The obtained radiotracer was successfully employed in the imaging of CCK-2R-expressing xenograft tumor in mice, but further structural studies are needed for enhancing receptor affinity and biodistribution. Additionally, the presented results are particularly noteworthy since the ability of a targeting probe to image cancers has been demonstrated using human cells expressing physiological levels of CCK-2R instead of transfected cells like the majority of the studies on the topic so far.

## Figures and Tables

**Table 1:** Predicted properties for Z-360 and IP-001. W-LogP: Wildman’s logP; TPSA: topological polar surface area; GI: gastrointestinal; BBB: blood brain barrier; ESOL: Estimated aqueous SOLubility; P-gp: P-glycoprotein; CYP: P-cytochrome.

	Z-360	IP-001
*W*-LogP	2.39	−3.22
TPSA (*Å*^2^; pH = 7.4)	121.88	356.36
logS (ESOL)	−5.49 (moderately soluble)	−1.79 (very soluble)
GI absorption	high	low
BBB permeant	no	no
P-gp substrate	yes	yes
CYP1A2 inhibitor	no	no
CYP2C19 inhibitor	no	no
CYP2C9 inhibitor	yes	no
CYP2D6 inhibitor	yes	no
CYP3A4 inhibitor	yes	no

**Table 2:** Amount of intact [^111^In]In-IP-001 over time in different media (*n* = 3; mean ± SD). The percentages were computed by means of radio-TLC (thin-layer chromatography) or RP-HPLC analyses. HEPES: 4-(2-hydroxyethyl)-1-piperazineethanesulfonic acid.

Stability (%)	4 h	8 h	24 h	72 h	120 h
NaCl 0.9%	98 ± 2	98 ± 2	97 ± 2	97 ± 2	97 ± 2
HEPES (0.1 M)	98 ± 2	97 ± 3	97 ± 3	96 ± 1	96 ± 3
EDTA (0.1 M)	98 ± 1	98 ± 1	96 ± 2	96 ± 3	96 ± 3
Human serum	98 ± 2	96 ± 3	95 ± 3	94 ± 2	93 ± 1
Human blood	98 ± 2	95 ± 1	95 ± 2	93 ± 1	93 ± 2

## Data Availability

Raw data are available at the following link: https://drive.google.com/drive/u/1/folders/1ohexfcWTNlfLvz5n42_VpdiXBGlh8DlL.

## Supplementary Materials

The following are available online: Figure S1: ^1^H NMR spectrum of **3** Figure S2: ^1^H NMR spectrum of **4**; Figure S3: ^1^H NMR spectrum of **6**; Figure S4: ^1^H NMR spectrum of **7**; Figure S5: ^1^H NMR spectrum of **8**; Figure S6: ^1^H NMR spectrum of IP-001; Figure S7: HRMS analysis of IP-001; Figure S8: Representative RP-HPLC chromatograms of free [^111^In]In^3+^ (**A**) and [^111^In]In-IP-001 (**B**); Figure S9: Representative radio-TLC chromatograms of free-[^111^In]In^3+^ (**A**) and [^111^In]In-IP-001 (**B**); Figure S10. ^1^H NMR spectrum of the compound due to the cyclization of the succinyl-group of compound **2**.

## Sample Availability

Samples of the compound IP-001 are available from the authors.

## Abbreviations

Cholecystokinin-2 (CCK-2), cholecystokinin-2 receptor (CCK-2R), nastorazepide (Z-360), cholecystokinin-1 receptor (CCK-1R), 1,4,7,10-Tetraazacyclododecane-1,4,7,10-tetraacetic acid (DOTA), 1,1′-carbonyldiimidazole (CDI), *tert*-butoxycarbonyl (Boc-), trifluoroacetic acid (TFA), dichloromethane (DCM), fluorenylmethoxycarbonyl (Fmoc-), dimethylformamide (DMF), nuclear magnetic resonance (NMR), high-resolution mass spectrometry (HRMS), Wildman’s logP (W-logP), topological polar surface area (TPSA), predicted solubility (log scale) computed according to the Estimated aqueous SOLubility (ESOL) model (LogS (ESOL)), gastrointestinal (GI), blood brain barrier (BBB), P-glycoprotein (P-gp), P-cytochrome (CYP), solid phase extraction (SPE), radiochemical purity (RCP), high-performance liquid chromatography (HPLC), thin-layer chromatography (TLC), 4-(2-hydroxyethyl)-1-piperazineethanesulfonic acid (HEPES), ethylenediaminetetraacetic acid (EDTA), acetonitrile (MeCN), single photon emission tomography (SPECT), computed tomography (CT), tumor to muscle (T/M), tumor to blood (T/B), maximum intensity projection (MIP), injected dose per cube centimeter (ID/cc), tetramethylsilane (TMS), matrix-assisted laser desorption/ionization time of flight (MALDI TOF/TOF), triethylamine (TEA), ethyl acetate (EtOAc), benzotriazol-1-yloxytris(dimethylamino)phosphonium hexafluorophosphate (BOP), human serum (HS), human blood (HB), 4′,6-diamidino-2-phenylindole (DAPI), phosphate buffer saline (PBS), maximum-likelihood expectation-maximization (MLEM), and iterative image space reconstruction algorithm (ISRA).
