# Supplementary material for: Preliminary Study of a 1,5-Benzodiazepine-Derivative Labelled with Indium-111 for CCK-2 Receptor Targeting
Source: Molecules. 2021 Feb 9;26(4):918. doi: 10.3390/molecules26040918 (PMC7916174; doi:10.3390/molecules26040918)
Supplement: Supplementary file 1 [file molecules-26-00918-s001.pdf]

Preliminary study of a 1,5-benzodiazepine-derivative labelled with indium-111 for CCK-2 receptors targeting.

Marco Verona[1], Sara Rubagotti[2], Stefania Croci[3], Sophia Sarpaki[4], Francesca Borgna[1], Marianna Tosato[5], Elisa Vettorato[1], Giovanni Marzaro[1,6], Francesca Mastrotto[1,6] and Mattia Asti[2,*]

[1] Department of Pharmaceutical Sciences, University of Padova, via Marzolo 5, 35131 Padova, Italy.
[2] Radiopharmaceutical Chemistry Section, Nuclear Medicine Unit, AUSL-IRCCS di Reggio Emilia, Viale Risorgimento 80, 42122 Reggio Emilia, Italy.
[3] Clinical Immunology, Allergy, and Advanced Biotechnologies Unit, Diagnostic Imaging and Laboratory Medicine Department, AUSL-IRCCS di Reggio Emilia, Viale Risorgimento 80, 42122 Reggio Emilia, Italy.
[4] Bioemtech, Lefkippos Attica Technology Park N.C.S.R. "DEMOKRITOS" Patr. Gregoriou E & 27 Neapoleos Str. 15341 Ag. Paraskevi, Athens, Greece.
[5] Department of Chemical Sciences, University of Padova, via Marzolo 1, 35131 Padova, Italy
[6] Italian Institute of Nuclear Physics, Legnaro National Laboratories, Viale dell'Università 2, 35020 Legnaro (Padova), Italy.

* Correspondence: asti.mattia@ausl.re.it; Tel.: +39-0522-296766

1. List of contents

- **Figure S1.** ^{1}H NMR spectrum of **3**
- **Figure S2.** ^{1}H NMR spectrum of **4**
- **Figure S3.** ^{1}H NMR spectrum of **6**
- **Figure S4.** ^{1}H NMR spectrum of **7**
- **Figure S5.** ^{1}H NMR spectrum of **8**
- **Figure S6.** ^{1}H NMR spectrum of **IP-001**
- **Figure S7.** HRMS analysis of **IP-001**
- **Figure S8.** Representative RP-HPLC chromatograms of free [^{111} In]In^{3+} (**A**) and [^{111}In]In-IP-001 (**B**).
- **Figure S9.** Representative radio-TLC chromatograms of free-[^{111}In]In^{3+} (**A**) and [^{111}In]In-IP-001 (**B**).
- **Figure S10.** ^{1}H NMR spectrum of the compound due to the cyclization of the succinyl- group of compound **2.**

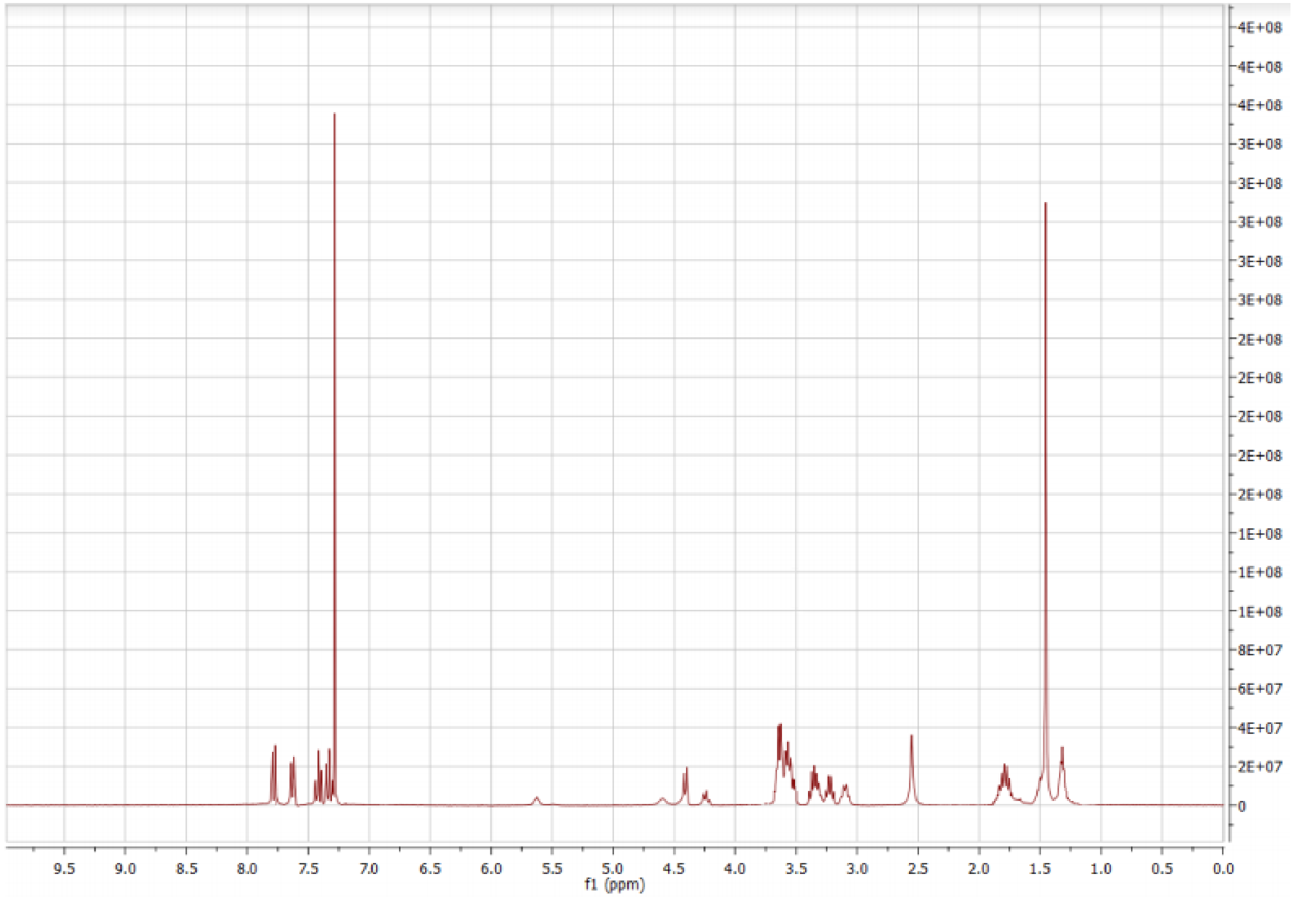

Figure S1. ^{1}H-NMR spectrum of **3** in $CDCl_3$ at 25 °C.

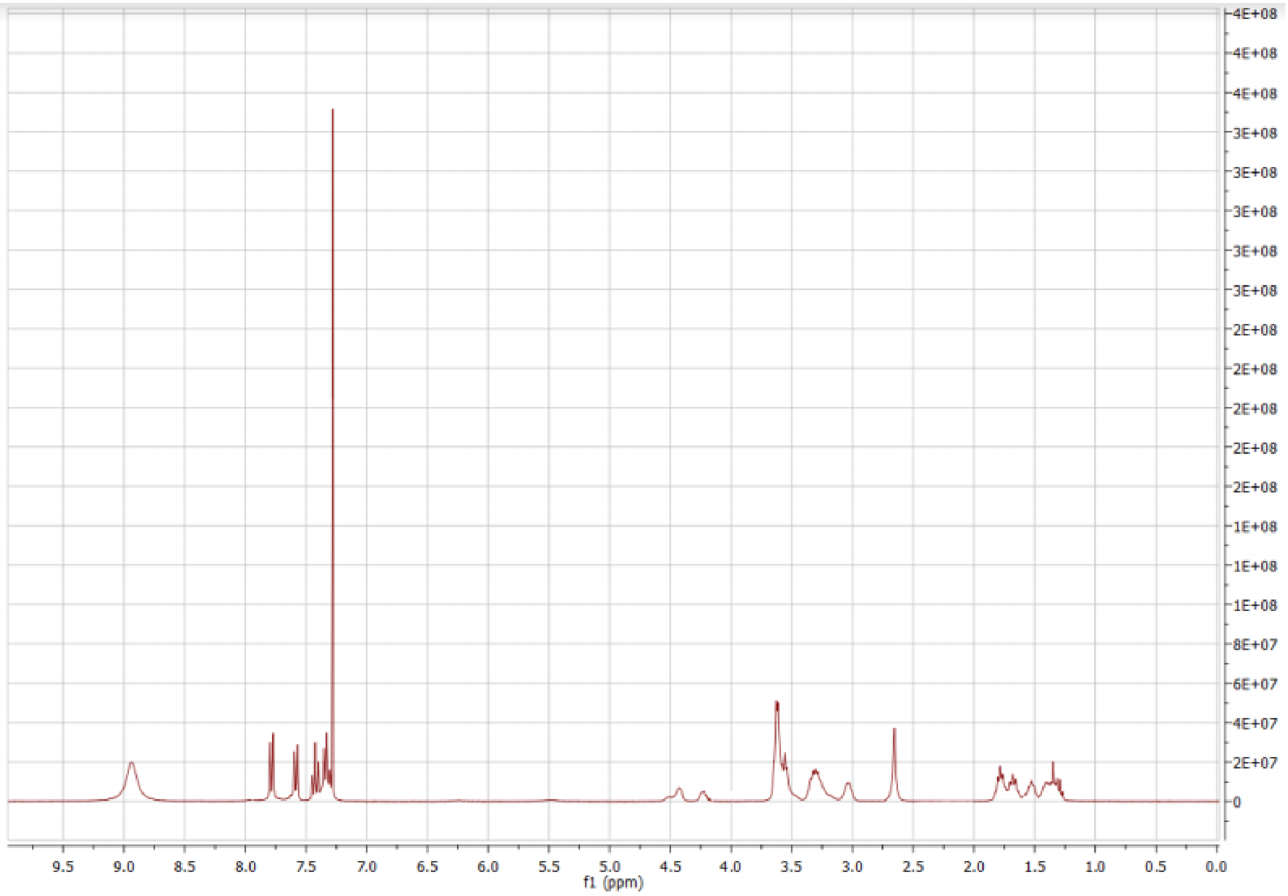

Figure S2. ^{1}H-NMR spectrum of **4** in $CDCl_3$ at 25 °C.

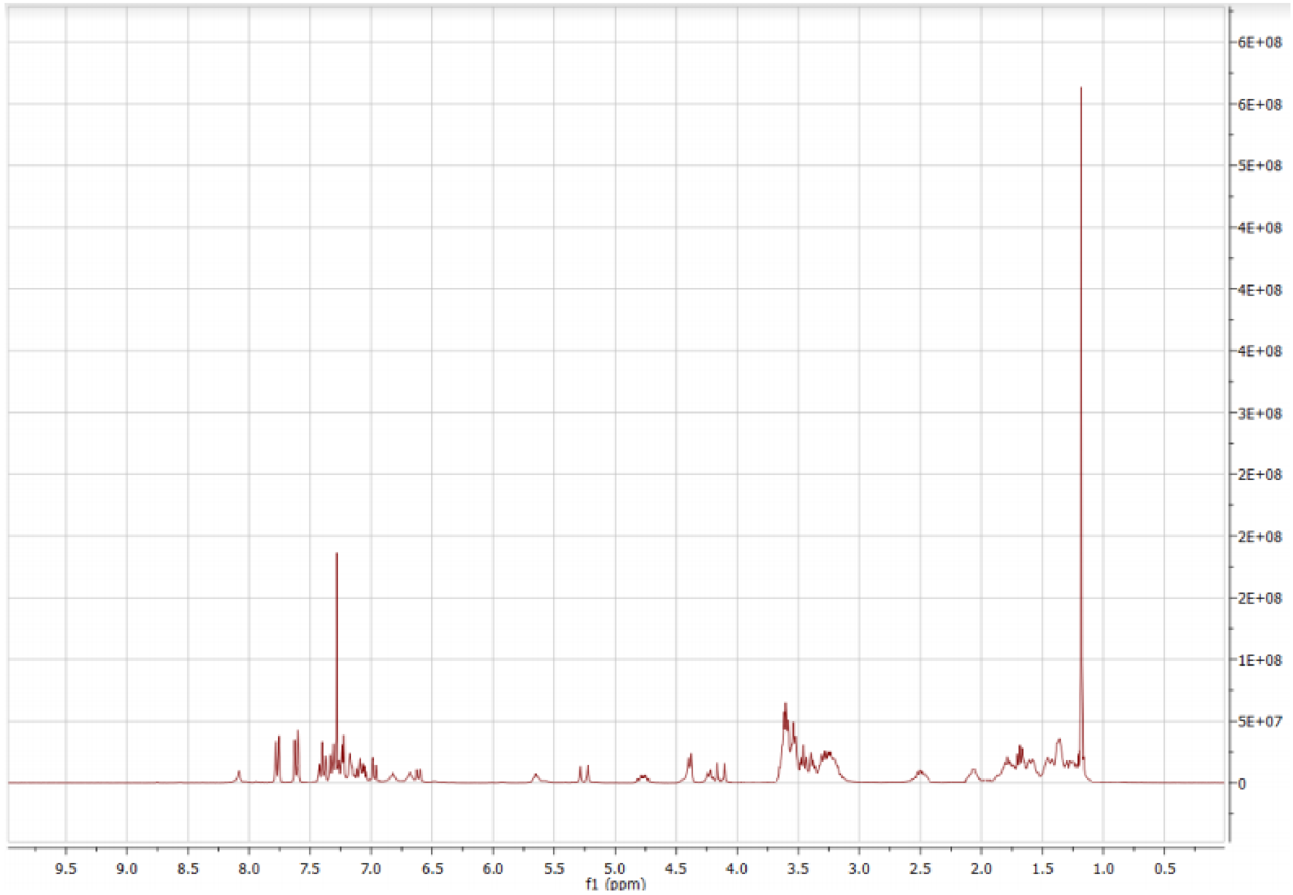

Figure S3. ^{1}H-NMR spectrum of **6** in $CDCl_3$ at 25 °C.

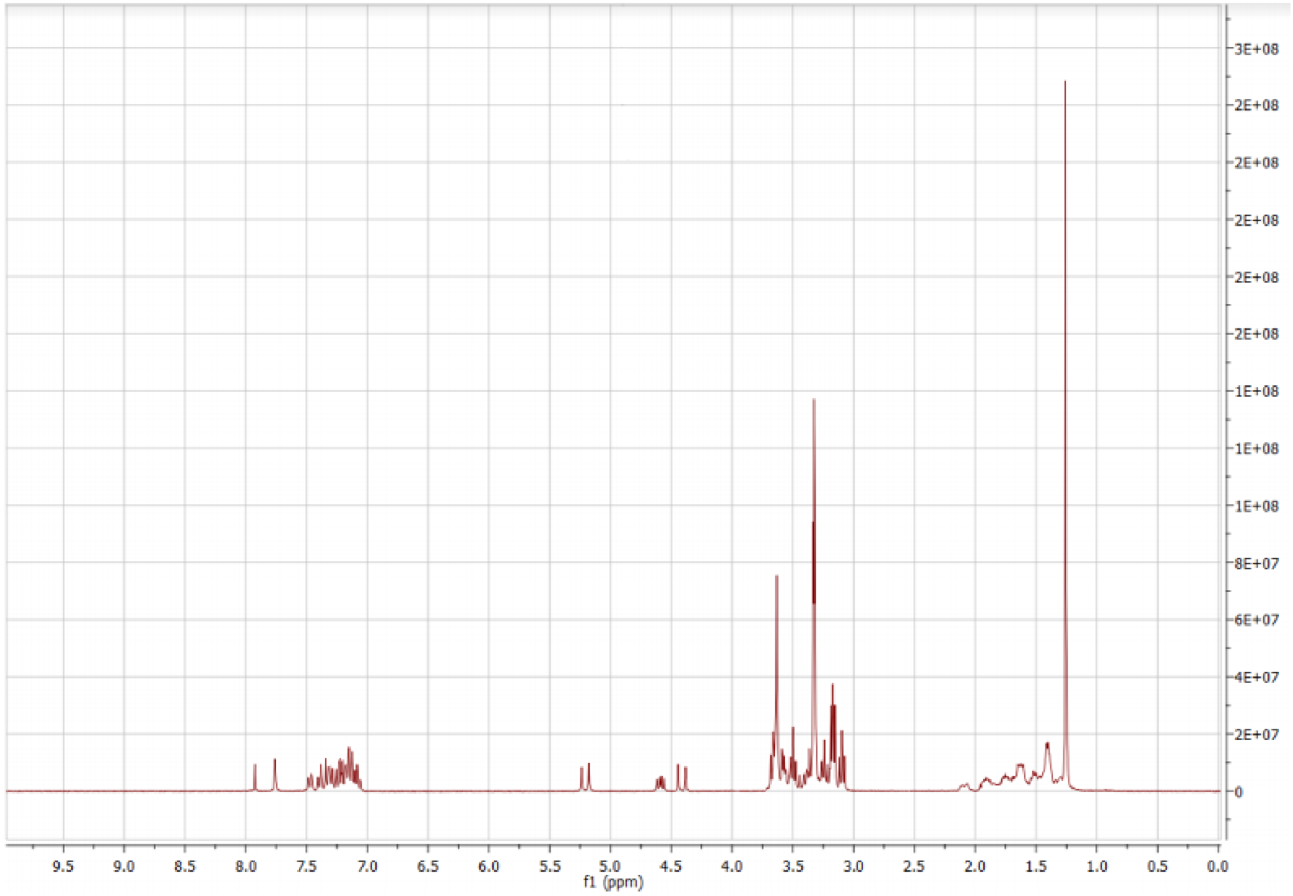

Figure S4. ^{1}H-NMR spectrum of **7** in $CDCl_3$ at 25 °C.

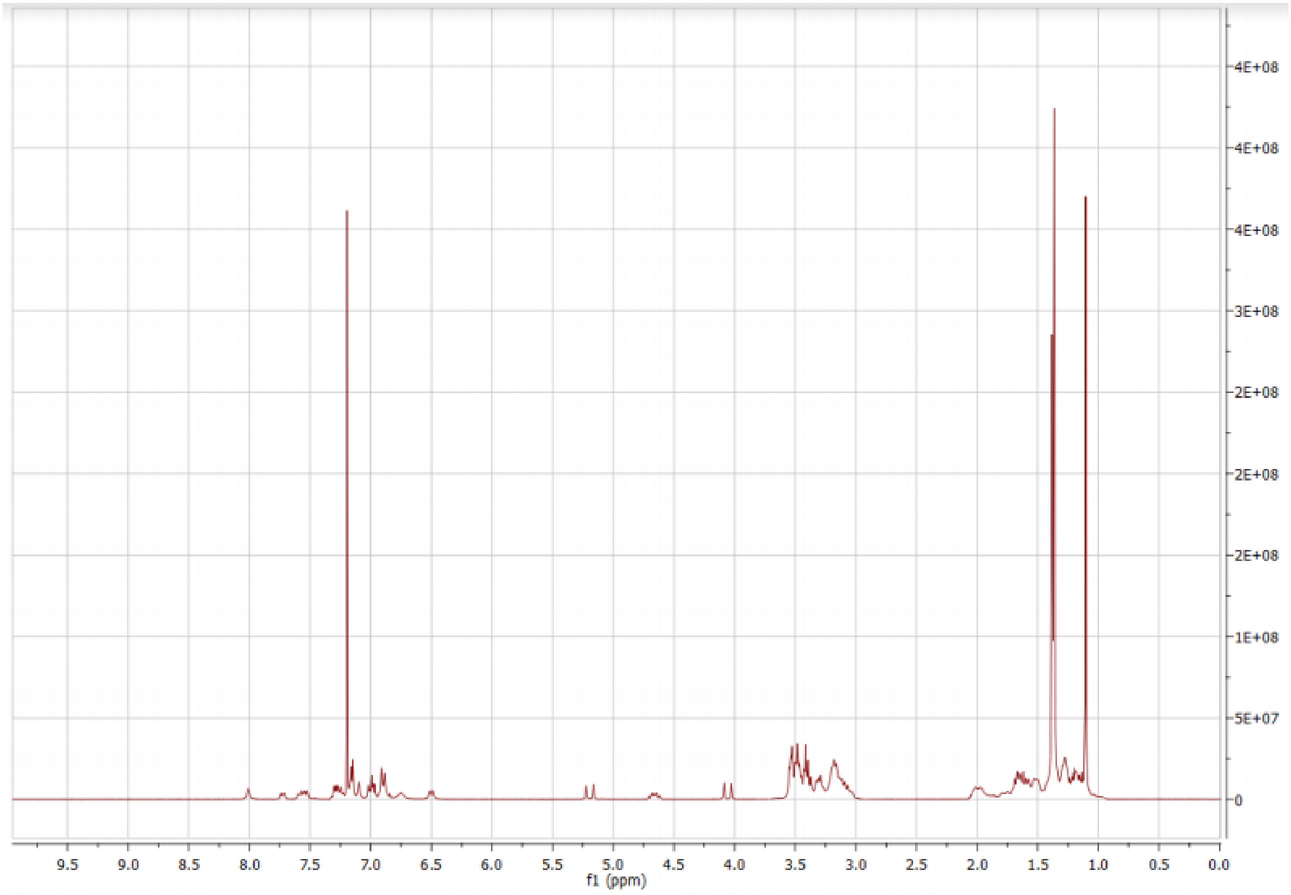

Figure S5. ^{1}H-NMR spectrum of **8** in $CDCl_3$ at 25 °C.

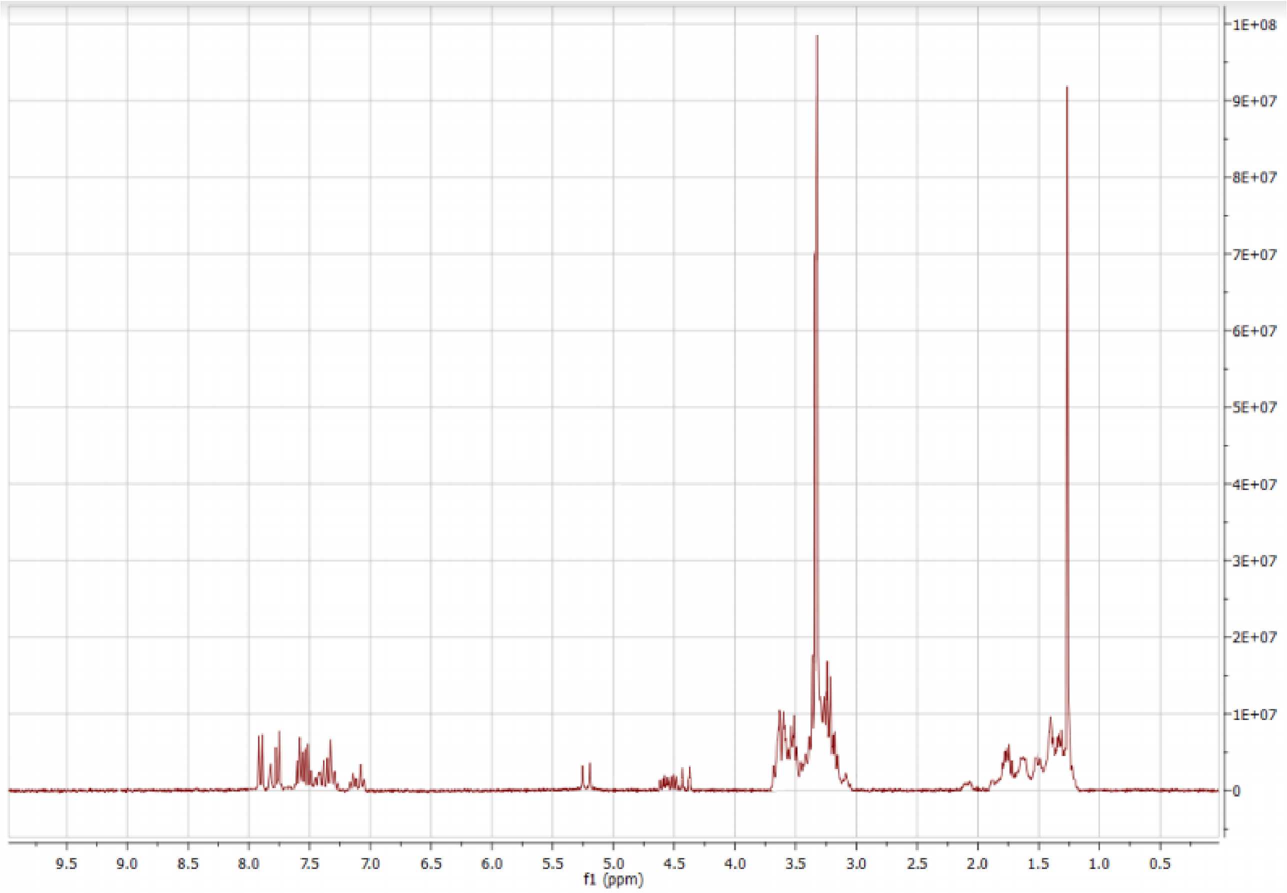

Figure S6. ^{1}H-NMR spectrum of **IP-001** in MeOD-d_4 at 25 °C.

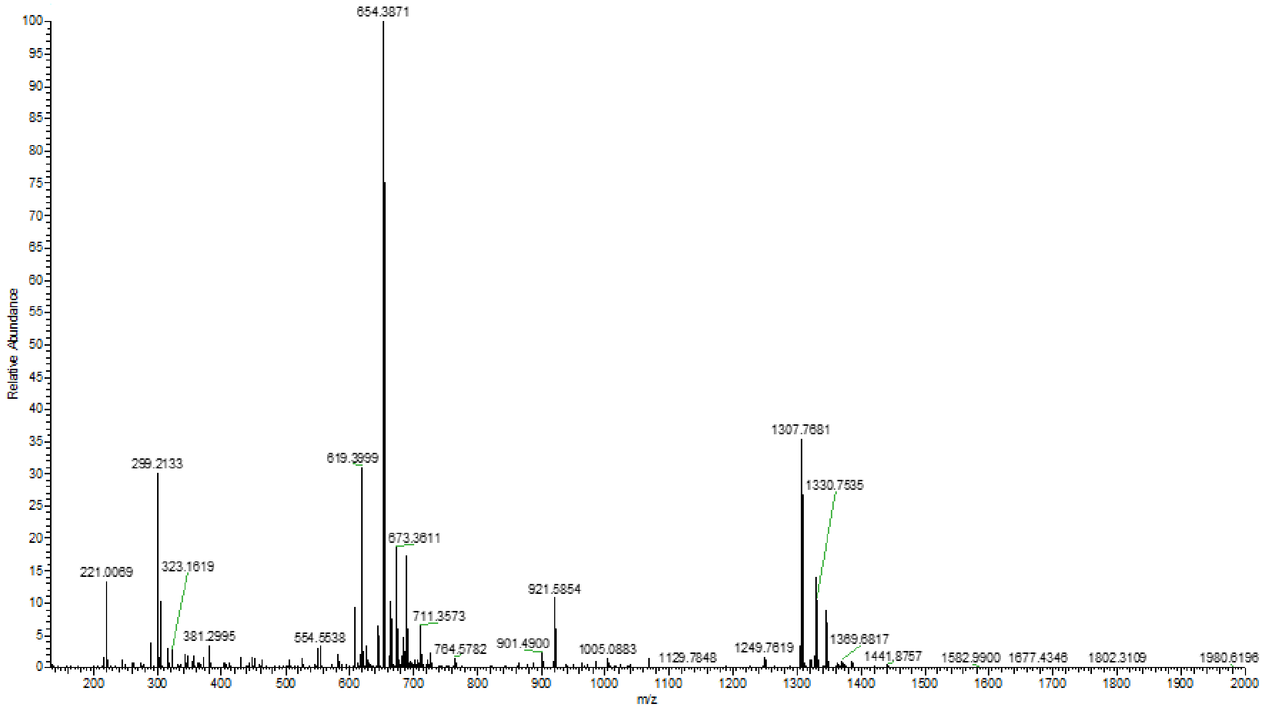

Figure S7. HRMS analysis of **IP-001.** m/z = 1307.76 [M+H$^+$]; m/z = 654.38 [M+2H$^+$]; m/z = 921.58 [M-DOTA]; m/z = 619.39 [M+H$^+$ of **7**, originating from fragmentation of **IP-001**].

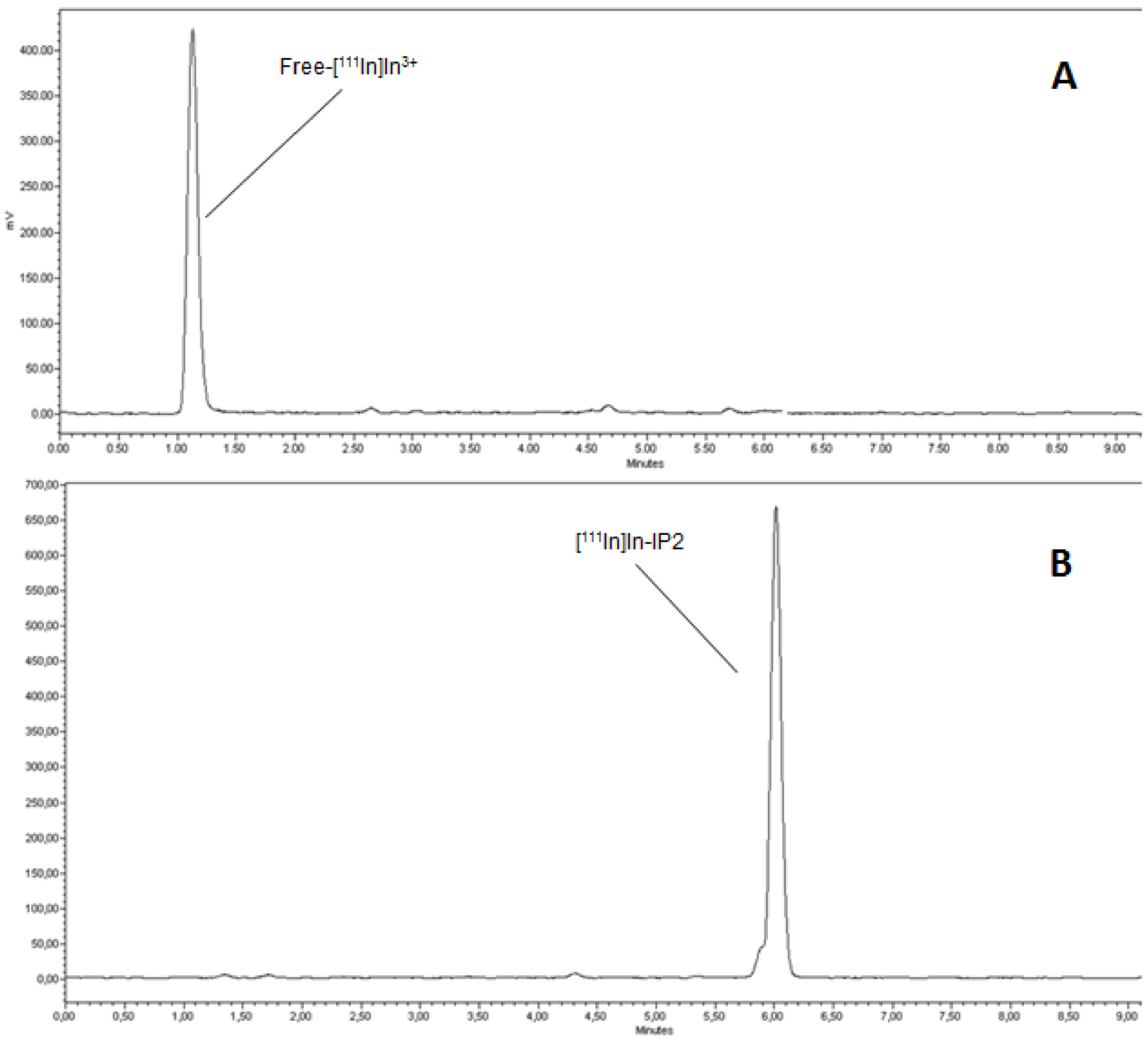

Figure S8. Representative RP-HPLC chromatograms of free $[^{111}\text{In}]\text{In}^{3+}$ (**A**) and $[^{111}\text{In}]$In-IP-001 (**B**). R_t: free-$[^{111}\text{In}]\text{In}^{3+}$ = 1.1 minutes; $[^{111}\text{In}]$In-IP-001 = 6.0 minutes.

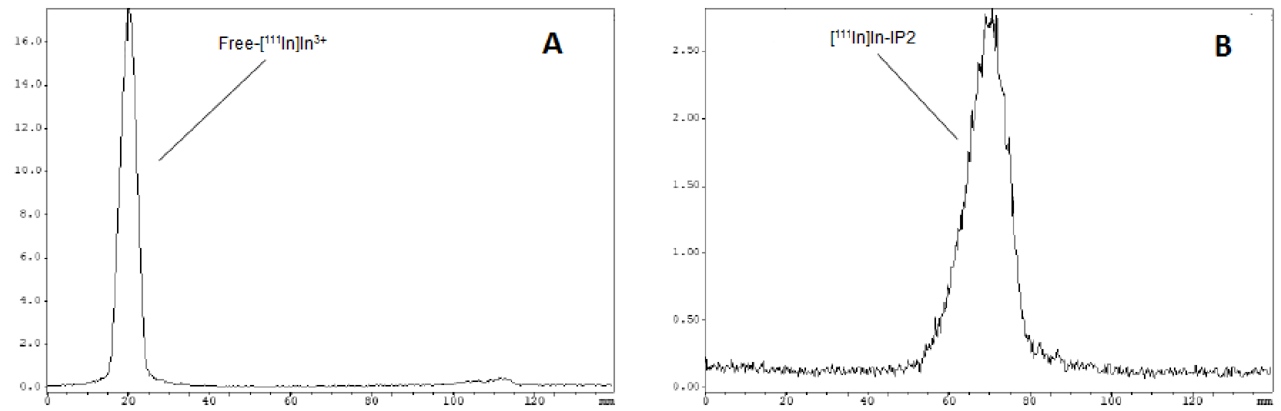

Figure S9. Representative radio-TLC chromatograms of free-[^{111}In]In^{3+} (**A**) and [^{111}In]In-IP-001 (**B**).

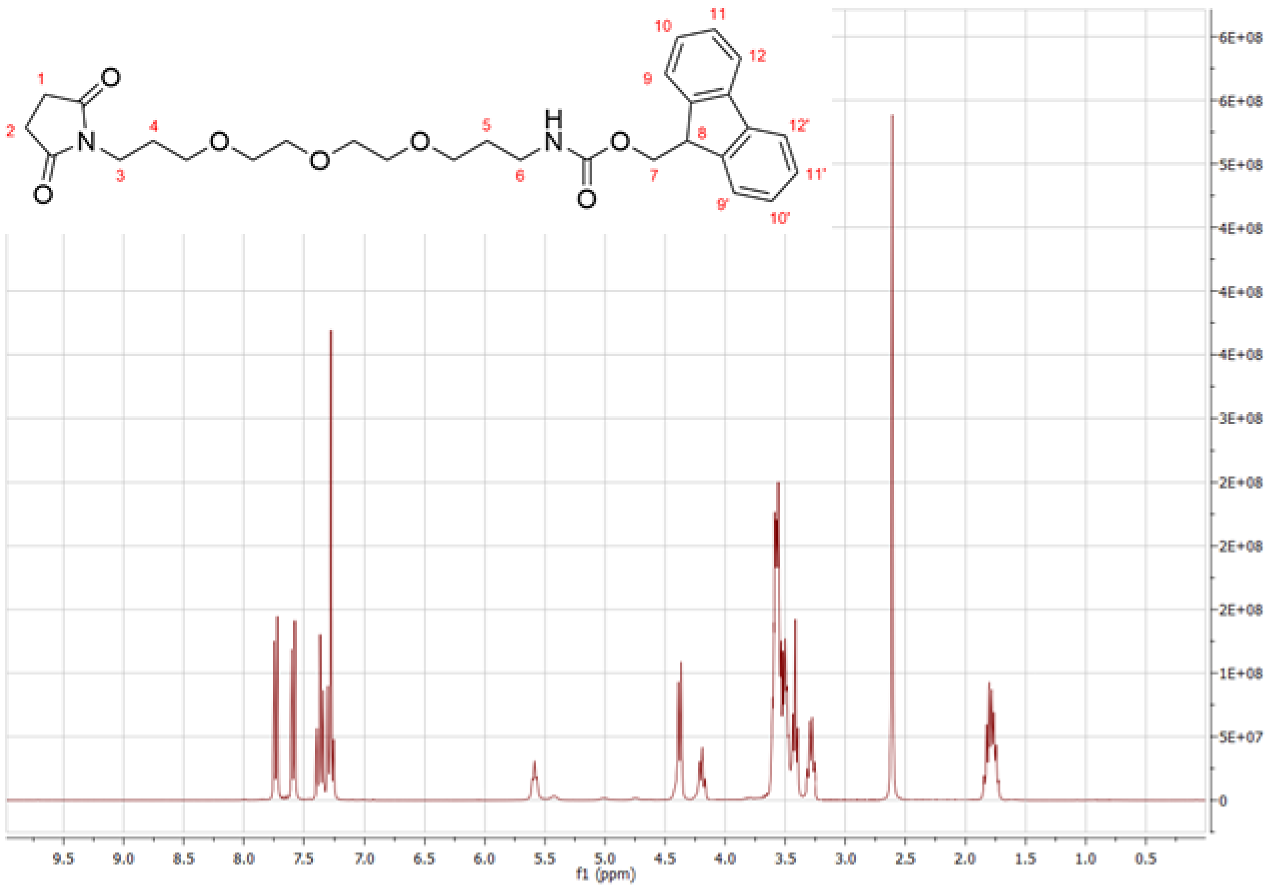

Figure S10. ^{1}H-NMR spectrum in $CDCl_3$ of the compound due to the cyclization of the succinyl- group of compound **2.** ^{1}H-NMR (400 MHz, $CDCl_3$) δ ppm 7.71 (d, *J*=7.4, 2H); 7.57 (d, *J*=7.4, 2H); 7.35 (t, *J*=7.3, 2H); 7.36 (t, *J*=7.3, 2H); 5.57 (t, *J*=5.4, 1H); 4.42-4.32 (m, 2H); 4.22-4.14 (m, 1H); 3.60-3.44 (m, 12H); 3.39 (t, *J*=6.4, 2H); 3.26 (q, *J*=6.2, 2H); 2.59 (s, 4H); 1.84-1.70 (m, 4H).
